# Deep Learning‐Powered Scalable Cancer Organ Chip for Cancer Precision Medicine

**DOI:** 10.1002/advs.202516660

**Published:** 2026-02-03

**Authors:** Yu‐Chieh Yuan, Beibei Xu, Jenna McCormack, XuHai Huang, Jingzhe Ma, Thomas Marshall, Yacong Sun, Hardeep Singh, Alyssa Fanelli, Gauri Kulkarni, Ji Hye Seo, Paige Gilbride, Bing Wei, Bo Wang, Yanyan Liu, Fei Ma, Lin Zhou, Shuyang Wang, Xiaohua Qian, Zhiyong Xie, Polina Golland, Longlong Si, Yu Shrike Zhang, Xin Xie, Haiqing Bai

**Affiliations:** ^1^ Xellar Biosystems Boston Massachusetts USA; ^2^ CAS Key Laboratory of Quantitative Engineering Biology Shenzhen Institute of Synthetic Biology Shenzhen Institute of Advanced Technology Chinese Academy of Sciences Shenzhen China; ^3^ Department of Molecular Pathology Henan Cancer Hospital Zhengzhou Henan China; ^4^ Henan Key Laboratory of Molecular Pathology Zhengzhou Henan China; ^5^ Department of Internal Medicine Henan Cancer Hospital Zhengzhou Henan China; ^6^ Department of General Surgery Henan Cancer Hospital Zhengzhou Henan China; ^7^ Henan Academy of Innovations in Medical Science Zhengzhou Henan China; ^8^ Department of Pathology School of Basic Medical Sciences Fudan University Shanghai China; ^9^ Computer Science and Artificial Intelligence Laboratory Massachusetts Institute of Technology Cambridge Massachusetts USA; ^10^ University of Chinese Academy of Sciences Beijing China; ^11^ Division of Engineering in Medicine Department of Medicine Brigham and Women's Hospital Harvard Medical School Cambridge Massachusetts USA

**Keywords:** drug screening, in silico staining, organ chip, precision medicine

## Abstract

Functional precision oncology complements genomic approaches by directly testing treatment options on patient‐derived models. However, existing platformssuch as patient‐derived xenografts (PDXs) and patient‐derived organoids (PDOs), face major barriers in clinical use due to technical challenges, including limited standardization, high costs, long assay times, scalability constraints, and incomplete recapitulation of the patient tumor microenvironment (TME). Here, we present a scalable, low‐cost Organ Chip (OC) platform fabricated entirely from thermoplastics via injection molding. Leveraging a patented channel geometry and surface treatment, the device achieves barrier‐free hydrogel confinement through capillary pinning without porous membranes, micropillars, or other barrier structures. This automation‐compatible platform supports tissue‐specific extracellular matrices and co‐culture through versatile perfusion modes, with robust imaging compatibility. We demonstrate its feasibility for drug sensitivity testing using multiple cell lines and patient‐derived primary cells, with imaging‐based phenotypic profiling for accurate quantification of drug responses, closely aligning with clinical outcomes. Additionally, we integrated a deep learning‐based image translation model that predicts fluorescence staining from bright‐field images. This approach enables longitudinal, label‐free phenotypic analysis with higher sensitivity than conventional endpoint staining. Together, this integrated cancer OC system overcomes key technical challenges and offers a promising framework for functional precision oncology through high‐throughput, patient‐relevant drug testing.

## Graphical abstract

This scalable, low‐cost Organ Chip platform, made via injection molding, uses capillary pinning for hydrogel confinement and supports versatile tissue coculture and robust imaging. Deep learning enables label‐free, sensitive phenotypic analysis. Demonstrated with multiple cancer models, it aligns closely with clinical outcomes, advancing high‐throughput, patient‐relevant drug testing for functional precision oncology.

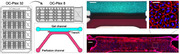

## 1 Introduction

The passage of the United States Food and Drug Administration (FDA) Modernization Act 2.0 [1] and the introduction of Act 3.0 [2] have catalyzed the integration of advanced in vitro models during drug development, notably in the challenging field of oncology, with a less than 5% success rate [3]. Meanwhile, patient‐derived models have emerged as promising tools to capture tumor heterogeneity and tailor treatments based on functional drug responses from a patient's own tumor cells, offering a practical complement to sequencing‐based precision oncology with broader applicability across cancer types and therapies [4].

Among various patient‐specific models, patient‐derived xenografts (PDXs) have been widely adopted due to their ability to maintain the 3D structure and cellular heterogeneity of human tumors in vivo. However, their applications are constrained by high costs, long preparation durations, ethical concerns, and a lack of immune system representation due to their reliance on immunodeficient murine hosts [4]. In contrast, patient‐derived organoids (PDOs) offer greater accessibility and retain their original architectures and genetic compositions in patients [5]. Nevertheless, the inability of PDO models to mimic the TME with high fidelity, such as the absence of critical stromal and vascular components, may impede a full understanding of tumor dynamics and compromise the accuracy of predicting drug sensitivities [4, 6]. Additional bottlenecks limiting their clinical utility include a suboptimal success rate of PDO generation due to an insufficient number of viable tumor cells, a lack of standardized assays and a predefined threshold to ensure reproducibility across labs, and a lengthy assay turnaround that typically exceeds 2 weeks [4, 6].

By using engineering strategies, such as microfabrication, to create geometries, perfusable channels, and dynamic flows, the OC technology holds the promise to simulate the microarchitectures and functions of human tissues and organs with greater human relevance and translational potential, offering a wide range of applications in understanding disease mechanisms, assessing drug toxicity and efficacy, and advancing personalized medicine [7, 8]. Cancer chips created using commercial systems or custom‐built devices have been utilized to study tumorigenesis at various stages and adopted for testing cancer therapies [9, 10].

Despite their potential, OC designs are often limited by complexity, low device yield, and high fabrication costs, restricting their translation beyond research settings [11, 12]. To address this, several commercial platforms and academic prototypes provide high‐throughput OC solutions [13, 14, 15, 16, 17, 18, 19, 20], generally categorized as membrane‐based or hydrogel‐patterning‐based strategies to compartmentalize tissues [21]. In membrane‐based OCs, porous synthetic barriers separate adjacent channels, facilitating transport studies and robust perfusion but often at the expense of direct cell‐cell or matrix‐cell contact [16, 17, 18]. In contrast, hydrogel‐patterning‐based OCs allow direct tissue‐tissue interactions across compartments, better mimicking native interfaces. However, most designs still rely on physical barriers such as micropillars, micro‐posts, or phase guides to confine hydrogel [13, 14, 15, 22, 23]. These geometric constraints, while effective, can introduce fabrication complexity that may impede fabrication scalability, potential risk of diffusion artifact and material autofluorescence, and incomplete tissue contact. Thus, despite progress, a fundamental trade‐off remains between robust hydrogel confinement, design scalability, and physiological fidelity.

In parallel, fluorescence microscopy has been the cornerstone for visualizing and quantifying drug responses, but poses several challenges, including labor‐intensive sample preparation, phototoxicity, potential alteration of cellular behaviors, and high costs [24]. Emerging label‐free bright‐field (BF) imaging, combined with deep learning‐based in silico staining approaches have shown promise for organoid analysis [24, 25, 26, 27, 28]. However, such methods have yet to be meaningfully deployed within the OC field, particularly for high‐throughput drug testing, where stable, fast, and accurate predictions are essential for real‐world applications [29, 30].

To overcome these technical limitations, we describe a highly scalable, cost‐efficient cancer OC platform fabricated entirely from thermoplastics using injection molding and thermal bonding. Distinct from prior multichannel OC systems that rely on micropillars, phase guides, or membranes to isolate hydrogel in one channel [14, 31, 32], our patented channel design and surface functionalization strategy enable barrier‐free hydrogel confinement in interconnected compartments via spatially controlled capillary pinning. This configuration allows seamless hydrogel loading, unrestricted cell‐cell interactions across compartments, and versatile perfusion modes with robust manufacturability and user‐friendliness. Using such a platform, we conducted proof‐of‐concept drug testing studies across several critical cancer types to demonstrate its capability for reliable, high‐throughput therapeutic testing. Finally, by integrating label‐free BF imaging with deep learning‐based analysis, we enabled non‐invasive, longitudinal tracking of cellular responses, achieving higher sensitivity and accuracy in quantifying drug effects. Together, this integrated system demonstrates the utility for both clinical drug testing in cancer patients and preclinical high‐throughput screening, offering significant promise for accelerating therapeutic discovery and personalized treatment strategies.

## 2 Results

### 2.1 Development of a Scalable OC‐Plex Device

**FIGURE 1:**
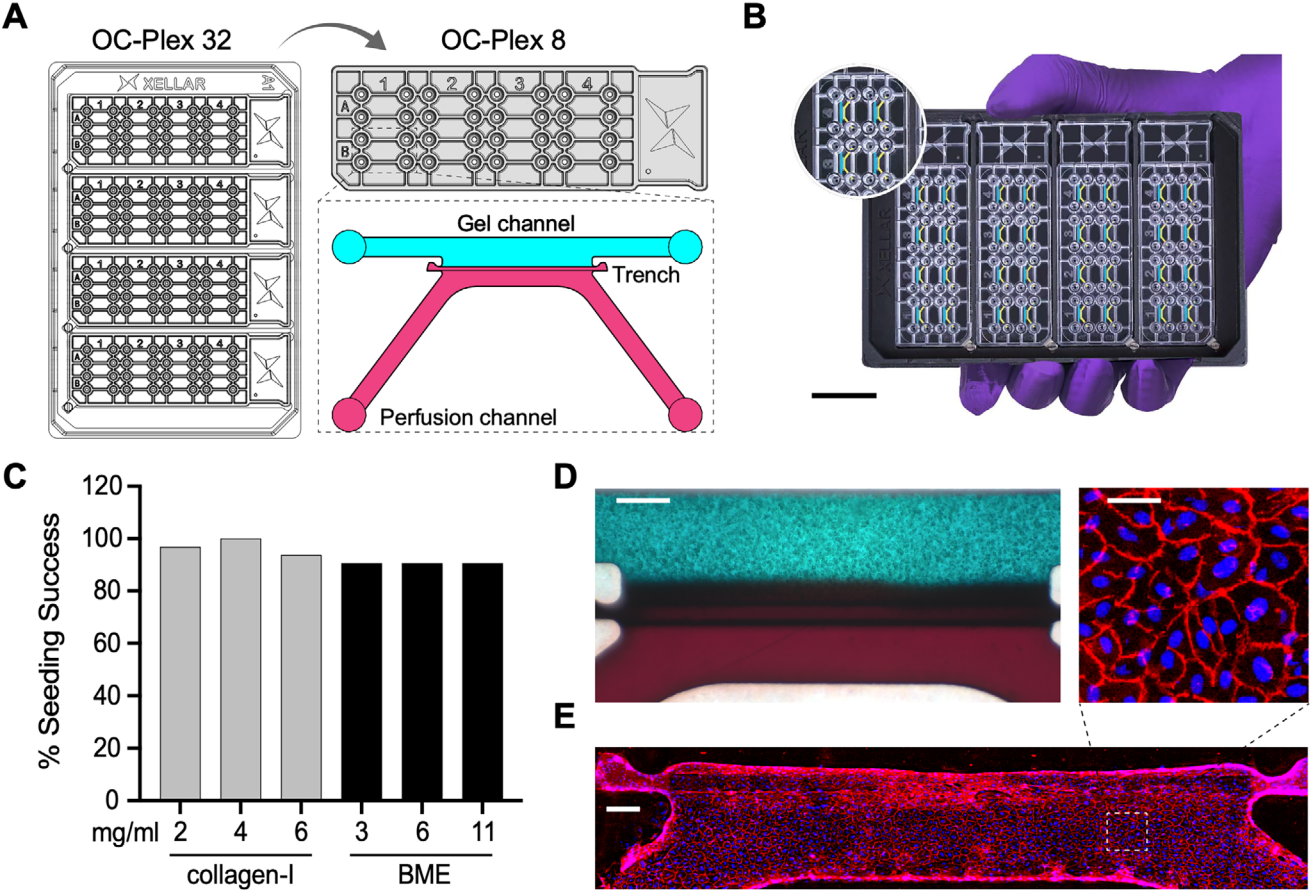
OC‐Plex platforms. (A) Diagram of the channel designs showing a gel channel and abutting perfusion channel in the OC‐Plex 8/32 device. (B) Photograph of the OC‐Plex 32 device in the carrier. Each device contains 8 chips; 4 devices can be placed in a stainless‐steel carrier for handling or imaging. Scale bar: 25 mm. (C) Percentage of seeding success for col‐1 gel and BME at indicated concentrations. *n* = 32 per group. (D) Representative images showing cells cultured in the gel channel and medium in food dye diffusion from the perfusion channel to the gel channel, forming a gradient. Scale bars: 400 µm. (E) Immunofluorescent staining of VE‐cadherin (red) and Hoechst nuclear staining (blue) of HUVECs in the perfusion channel. Scale bar: 200 µm (chip view) and 50 µm (zoom‐in).

A desirable microfluidic device for functional oncology requires a physiologically relevant cell culture system with advanced microfluidics for co‐culture. This is critical to accurately mimic the complex TME and to evaluate the effectiveness of therapeutics. The system should also adopt biocompatible materials for scalability and cost‐efficiency. To address these requirements, we designed a fully thermoplastic microfluidic device (Figure 1A,B), which we termed OC‐Plex, manufactured by industrial‐scale injection molding and thermal bonding.

Within each OC‐Plex 8 device are 8 independent chips containing two channels – a gel channel for culturing cells embedded in 3D hydrogels and a perfusion channel for nutrient delivery, each having a width of 500 µm and a height of 250 µm (Figure 1A,B). These channels converge in a 3‐mm‐long and 250‐µm‐deep barrierless culture interface where hydrogel confinement is achieved not through physical structures (e.g., micropillars, membrane, or phase guides), but via capillary pinning at a patented geometrically expanded channel interface (Figure 1A). This is further supported by surface coating that results in a balanced channel surface hydrophilicity/hydrophobicity, allowing hydrogel or hydrogel/cell mixture to flow from one end of the gel channel to the other end, even though any barrier feature is absent between the two channels (Video S1).

Gel filling test revealed a filling success (defined as no spillover from the gel channel to the perfusion channel or partial flow across the gel channel) rate of above 90% for Matrigel and collagen I (col‐1) under different concentrations (Figure 1C). This design permits the establishment of physiologically relevant gradients of oxygen and nutrients across the two channels (Figure 1D; Figure S1). In addition, the perfusion channel can be seeded with endothelial cells to form a vascularized tubule or interfaced with the gel channel for co‐culture (Figure 1E; Figure S2A–C).

To enable higher throughput, four independent OC‐Plex 8 devices can be grouped to form OC‐Plex 32 by placing them in carrier, which also serves as a microscopy adaptor that is compatible with most automated imaging systems (Figure 1A,B). The microchannels are interfaced with ports spaced following the ANSI/SLAS 4–2004 (R2012) format to ensure automation compatibility.

The OC‐Plex platform is optimized for cost‐efficient, scalable production, with fabrication methods that allow for rapid manufacturing and low material consumption. Compared to many existing academic and commercial organ‐on‐chip systems, which often involve complicated and labor‐intensive processes, OC‐Plex offers a more streamlined and economical alternative. In addition, OC‐Plex device accommodates flexible perfusion modes: devices can be connected with a peristatic pump for constant rate of flow or coupled with an injection‐molded polystyrene (PS) reservoir component to enable gravity‐driven flow on a rocker (Figure S2D,E).

### 2.2 OC‐Plex Supports Cancer Cell Growth and Drug Sensitivity Testing

**FIGURE 2:**
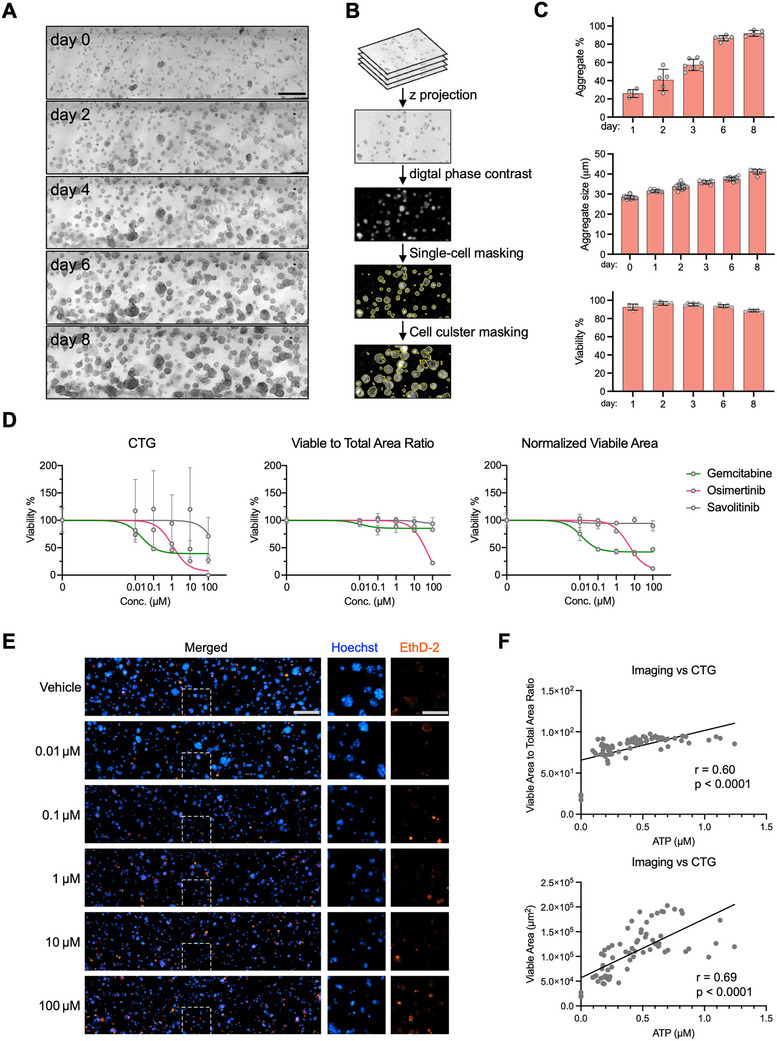
Device supports cancer cell growth. (A) Representative BF images showing single‐cell embedded BxPC‐3 growing for 8 days in the same OC‐Plex chip. Scale bar: 250 µm (B) Schematic illustrating analysis strategy for longitudinal imaging to quantify cell growth over time. BF Z‐stack images capturing the entire volume of the chips were compressed into Z‐projections for image‐processing and analyses. Each single cell or cell cluster is segmented as an object, indicated by a yellow contour. (C) Image analysis results for BxPC‐3 growth in OC‐Plex showing % of aggregates (top), aggregate size (middle), and viability (bottom) chips on 0, 1, 2, 3, 6, and 8 days after culture. Data show mean ± s.d.; *n* = 4–7 chips. (D) Drug dose response curves determined by CTG (left), viable to total cell ratio (middle), and viable area (right) after treatment with gemcitabine, gefitinib, and osimertinib for 48 h. All metrics are normalized relative to vehicle control (100%). Data show mean ± s.d.; *n* = 4 chips. (E) Representative images of chips treated with osimertinib at 0.01–100 µm for 48 h and then stained with Hoechst33342 and EthD‐2 for viability. The right two image columns are the zoomed‐in views of the regions in the corresponding gray panes. Scale bars: 250 µm (merged image overview) and 120 µm (zoomed‐in view on the right). (F) Correlation graphs comparing imaging results to CTG result. Each dot represents one chip.

To first demonstrate the feasibility of OC‐Plex for establishing robust and reproducible cancer OC models, we developed a pancreatic ductal adenocarcinoma (PDAC) on‐chip model using the BxPC‐3 cell line. BxPC‐3 were embedded in 4 mg/mL of col‐1 solution as single cells, loaded into OC‐Plex 8 devices in the gel channel and subsequently cultured for 8 days (Figure 2A). An image‐analysis pipeline was established to perform image segmentation and feature extraction from Z‐projections of the serial Z‐stacks of BF images (Figure 2B). Feature analysis revealed a gradual increase in the percentages of aggregates (Figure 2C top) and the object size (Figure 2C middle). Hoechst33342 and ethidium homodimer‐2 (EthD‐2) staining confirmed high degrees of cell viability throughout the culture period (Figure 2C bottom). Further testing of different seeding densities ranging from 0.3 × 10^6^ cells/mL to 5 × 10^6^ cells/mL, corresponding to 300 to 5000 cells per chip, revealed several phenotypic changes in cell morphology during on‐chip culture for 15 days, including cell aggregate‐formation, fusion, fission, and migration (Figure S3).

Similarly, aggregate‐formation was observed for the lung cancer cell line A549 when embedded in 100% Basement Membrane Extract (BME) (Figure S4A,B). In addition, cells maintained high viability when cultured under static condition with or without the reservoirs or under dynamic conditions with the reservoirs (Figure S4C,D). Notably, we noted more pronounced cell migration from the gel channel to the perfusion channel on day 8 under dynamic conditions, resulting in a smaller percentage of aggregates in the gel channel than under static condition (Figure S4C,D), likely due to high exchange rates of oxygen and nutrients and thus faster cell growth associated with medium perfusion. Lastly, the OC‐plex device also supported cell growth and spheroid‐formation for colon cancer cell lines HCT116 and HT‐29 (Figure S5). Collectively, these data strongly indicate that our device effectively supports cell growth, spheroid formation, cell migration, and long‐term viability across diverse cancer types, various ECM conditions, and multiple modes of medium perfusion.

Next, we sought to demonstrate the practical utility of the OC‐Plex device in cancer drug testing. Sensitive and quantitative readouts are critical for a scalable cancer drug screening platform and imaging may represent a better readout for therapeutic assessment compared to traditional biochemical assays. To test this, BxPC‐3 were cultured on chip for 24 h and exposed to cancer drugs with 0.1% dimethyl sulfoxide (DMSO) as negative controls. After 48 h of drug treatment, viability staining was conducted, and whole‐chip images were acquired using an automated microscope. We also performed the CellTiter‐Glo (CTG) assay, the most widely used assay for assessing therapeutic responses in 3D cancer models by measuring the amount of cellular adenosine triphosphate (ATP) [33]. Importantly, we confirmed that the CTG assay could be performed following viability staining without a significant impact on ATP content, thus allowing a direct comparison of the two readouts (Figure S6A,B).

To establish the imaging assay and identify the best imaging feature for quantifying drug effects, we selected three cancer drugs: gemcitabine, gefitinib, and osimertinib and tested their responses in BXPC‐3 culture on‐chip. Gemcitabine is the standard first‐line therapy for pancreatic cancer, while epidermal growth factor receptor (EGFR) inhibitors, such as gefitinib and osimertinib, are two FDA‐approved drugs for non‐small‐cell lung cancer (NSCLC) that have drawn increased attention in treating pancreatic cancer with EGFR activation [34]. CTG results (Figure 2D) indicated that gemcitabine had a smaller half‐maximal inhibitory concentration (IC_50_), whereas EGFR inhibitors, especially osimertinib, resulted in greater inhibition at high concentrations. A total of 60 imaging metrics including intensity, shape, and texture features were ranked based on their Z’‐factors, a statistical index reflective of both the assay signal dynamic range and data variation [35] (Figure S6C). The two metrics with the highest Z’ scores, namely “Normalized Viable Area” and “Viable to Total Area Ratio”, were chosen to generate drug dose‐response curves. Compared to results from the CTG assay, these imaging metrics revealed a similar trend in ranking drug sensitivity but with much smaller data variations (Figure 2D).

Between the two imaging features, we observed that the “Viable to Total Area Ratio” feature is associated with smaller data variations and greater dose‐response dynamics compared to “Normalized Viable Area” (Figure 2D). While “Viable to Total Area Ratio” can mitigate variations due to seeding homogeneity, it may also neglect drug effects independent of cell death. This is exemplified by gemcitabine‐induced cell growth‐inhibition (Figure 2E), wherein higher concentrations led to smaller aggregate sizes without a significant increase in cytotoxicity (EthD‐2 positive signal). In sum, the normalized viable area represents a better feature overall in measuring drug effects. Consistently, we observed a higher correlation between ATP levels and viable areas than between ATP levels and “Viable to Total Area Ratio” (Figure 2F). In addition, we showed that EthD‐2 staining can be combined with other cell death markers, such as caspase‐3/7, to enable even greater sensitivity in measuring drug responses (Figure S7). These findings suggest that the OC‐Plex device, when combined with staining and imaging analysis, provides a quantitative and reproducible platform for evaluating therapeutic responses.

### 2.3 Cancer Drug Sensitivity Testing

**FIGURE 3:**
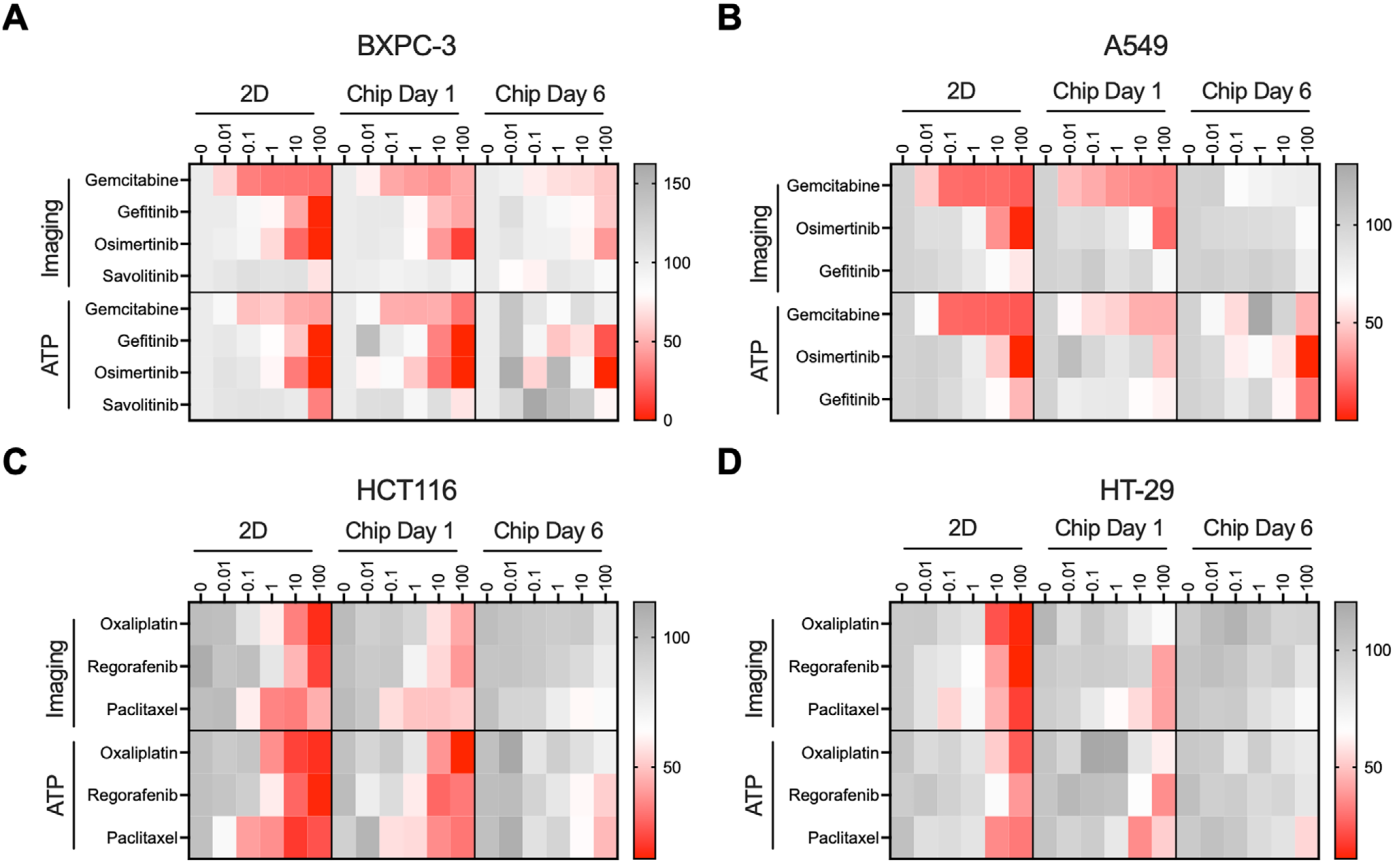
Drug sensitivity testing. Normalized dose‐dependent heatmaps of cell survival after different drug treatments in 2D and cancer OC models in BXPC‐3 (A), A549 (B), HCT116 (C), and HT‐29 cells. (D) Survival results were obtained by CellTiter‐Glo ATP assay and imaging assay (*n* = 4), respectively. 2D represents the 2D model; Chip Day1 represents the cells pre‐cultured for 1 day on the OC‐Plex device followed by the drug treatment; Chip Day7 represents cells pre‐cultured on OC‐Plex device for 7 days followed by dosing treatment. Red represents lower cell survival and better drug treatment, and gray represents higher cell survival and worse drug treatment.

We further compared the drug sensitivity profiles between short‐term and long‐term cultures to evaluate how culture duration influences therapeutic response. Specifically, we cultured cancer cells for short‐term (1 day) and long‐term (6–7 days) periods before treating the chips with cancer drugs for 48 h, with traditional 2D culture in 96‐well plates as controls. We initially focused on BxPC‐3 pancreatic cancer cells and tested gemcitabine, gefitinib, osimertinib, and savolitinib (a small molecule inhibitor of c‐MET used as a negative control). Analyses of drug IC_50_ and area under the curve (AUC) using imaging metrics revealed a consistent trend in drug sensitivity (Table S1). However, a decreased drug effect was observed on‐chip when dosed on day 7 compared to day‐1 dosing or 2D controls (Figure S8). This trend was also visualized in a heatmap comparing the percentage of inhibition across different models (Figure 3A). Consistent with previous analyses (Figure S6C), imaging outperformed the CTG assay with higher assay sensitivity and lower chip‐to‐chip variation.

We next tested the same set of drugs in the A549 lung cancer model. Imaging analyses confirmed that gemcitabine was the most sensitive drug, followed by gefitinib and osimertinib when drugs were dosed at day 1 after culture on chip (Figure S9A,B). Feature analyses confirmed that the same top imaging features selected for BxPC‐3 cells could be applied to A549 cells (Figure S9F–H). Again, a significantly decreased drug effect was observed on chip when dosed on day 7 compared to day‐1 dosing or 2D control (Figure 3B; Table S1).

For colorectal cancer, we selected representative drugs commonly used in colorectal cancer treatment: oxaliplatin, regorafenib, and paclitaxel [36, 37]. These drugs were tested in HCT116 and HT‐29 cells in a similar fashion. In general, significant differences in drug inhibition were observed between 2D and on‐chip cultures (Figure 3C,D; Table S1): even after 1‐day culture on chip, both HCT116 and HT‐29 cells had significantly reduced sensitivity compared with the 2D control. The sensitivity was further reduced when drug treatment was initiated 6 days after culture on chip. These results suggest that the 2D culture model may produce false‐positive results when predicting drug response in vitro, and that longer culture durations may offer a closer approximation to in vivo drug penetration, and thus, cancer cell sensitivity to drugs. Again, in this type of cancer model, imaging readouts had higher data stability and accuracy compared to the CTG method (Figures S10A and S11A).

Finally, we assessed automation compatibility of the OC‐Plex platform using A549 cells (Figure S12). Key workflows for drug testing were automated via an off‐the‐shelf liquid handler, yielding similar drug responses comparable to the manual workflow. However, automation reduced total handling time by approximately 70% for a 7‐day experiment, underscoring the scalability potential of the system.

### 2.4 Cancer Drug Sensitivity Testing Using Patient‐Derived Cells

**FIGURE 4:**
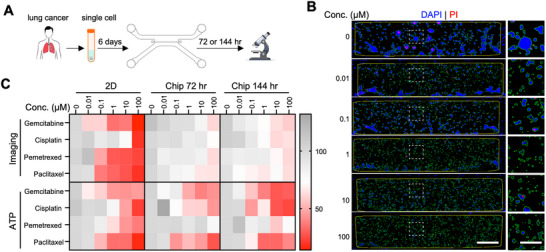
Cancer drug sensitivity testing using primary patient samples. (A) Schematic of the workflow for on‐chip cancer drug testing with patient‐derived samples. Cells were cultured on‐chip for 6 days, followed by drug treatment for either 3 or 6 days. (B) Representative images of gemcitabine treatment on‐chip, showing segmentation and classification of live and dead cells for quantifying drug effects. Scale bars: 200 µm (whole chip) and 50 µm (zoomed‐in view). (C) Heatmaps displaying dose‐response curves of cell viability from 2D culture and on‐chip cultures, measured via imaging or ATP readouts. Red indicates lower cell viability, while gray indicates higher cell viability. Pemetrexed, highlighted in red, shows a divergent response between 2D and on‐chip cultures.

To determine whether our cancer chip model could be used to predict clinical outcomes, we cultured freshly isolated cells from a patient with squamous NSCLC. We selected representative therapeutic drugs commonly used in the clinical treatment of lung cancer, including gemcitabine, pemetrexed, paclitaxel, and cisplatin [38], and treated lung chips with these drugs for 72 h or 144 h after cell culture on‐chip for 6 days (Figure 4A). A segmentation algorithm was applied to select regions of interest (ROIs) on the chip and subsequently used for classification of live or dead cells, which were used to determine cell viability (Figure 4B). In 2D culture, gemcitabine, pemetrexed, and paclitaxel showed comparable efficacy after 72 h of drug treatment, whereas the cells exhibited higher resistance to cisplatin (Figure S13A,B). However, a different drug sensitivity profile was observed on‐chip (Figure 4C; Table S1). Specifically, with a 72‐h dosing regimen, the cells were most sensitive to paclitaxel, followed by cisplatin, gemcitabine, and pemetrexed (Figure S13C,D). With extended drug dosing of 144 h, paclitaxel continued to show the strongest efficacy, and the efficacies of gemcitabine and cisplatin increased compared to the 72‐h dosing (Figure S13E,F). Pemetrexed efficacy, nonetheless, did not significantly increase with prolonged exposure. Unlike the 2D culture results, the lack of efficacy for pemetrexed on‐chip aligns with clinical trial findings that patients with squamous NSCLC do not benefit from pemetrexed and face a higher risk of certain adverse effects [39, 40]. Therefore, our results demonstrate that the OC‐Plex device can be used for the culture of patient‐derived cancer cells, and on‐chip drug testing may be more accurate in predicting drug efficacies in humans compared to 2D models.

To further evaluate the clinical relevance of the OC‐Plex platform, we tested whether the device supports functional immune–tumor interactions using patient‐derived gastric cancer organoids co‐cultured with activated human peripheral blood mononuclear cells (PBMCs). Following 5 days of co‐culture, organoids were treated with vehicle control, low‐dose (10 µg/mL), or high‐dose (200 µg/mL) PD‐1 inhibitor sintilimab. Longitudinal bright‐field and fluorescence imaging over 0–48 h revealed DiO‐labeled PBMCs progressively infiltrating the organoid structures, accompanied by sintilimab‐induced cytotoxicity characterized by structural disruption and reduced viability in a time‐ and dose‐dependent manner (Figure S14A). Propidium Iodide (PI)/Hoechst staining further confirmed increased cell death in drug‐treated conditions with the strongest response at 200 µg/mL (Figure S14B). These results demonstrate that OC‐Plex can model immune‐mediated antitumor responses in a patient‐derived context with quantitative evaluation of checkpoint inhibitor activity, further supporting its use for testing clinically relevant therapeutic modalities.

### 2.5 A Deep Learning‐Based Pipeline to Enable Label‐Free Phenotypic Analysis on Chip

Fluorescence microscopy is widely used to visualize specific (sub)cellular structures through targeted labeling, but its application requires advanced equipment and labor‐intensive sample preparation. In contrast, BF imaging offers a non‐invasive, label‐free alternative for capturing cellular morphology, enabling continuous monitoring of cellular changes over time [41]. We reasoned that, by mapping BF images to fluorescence‐equivalent images, it is possible to extract fluorescence‐specific information without physical staining. This mapping nonetheless, relies on training models to learn the relationships between BF and fluorescence image modalities using spatially registered image pairs.

**FIGURE 5:**
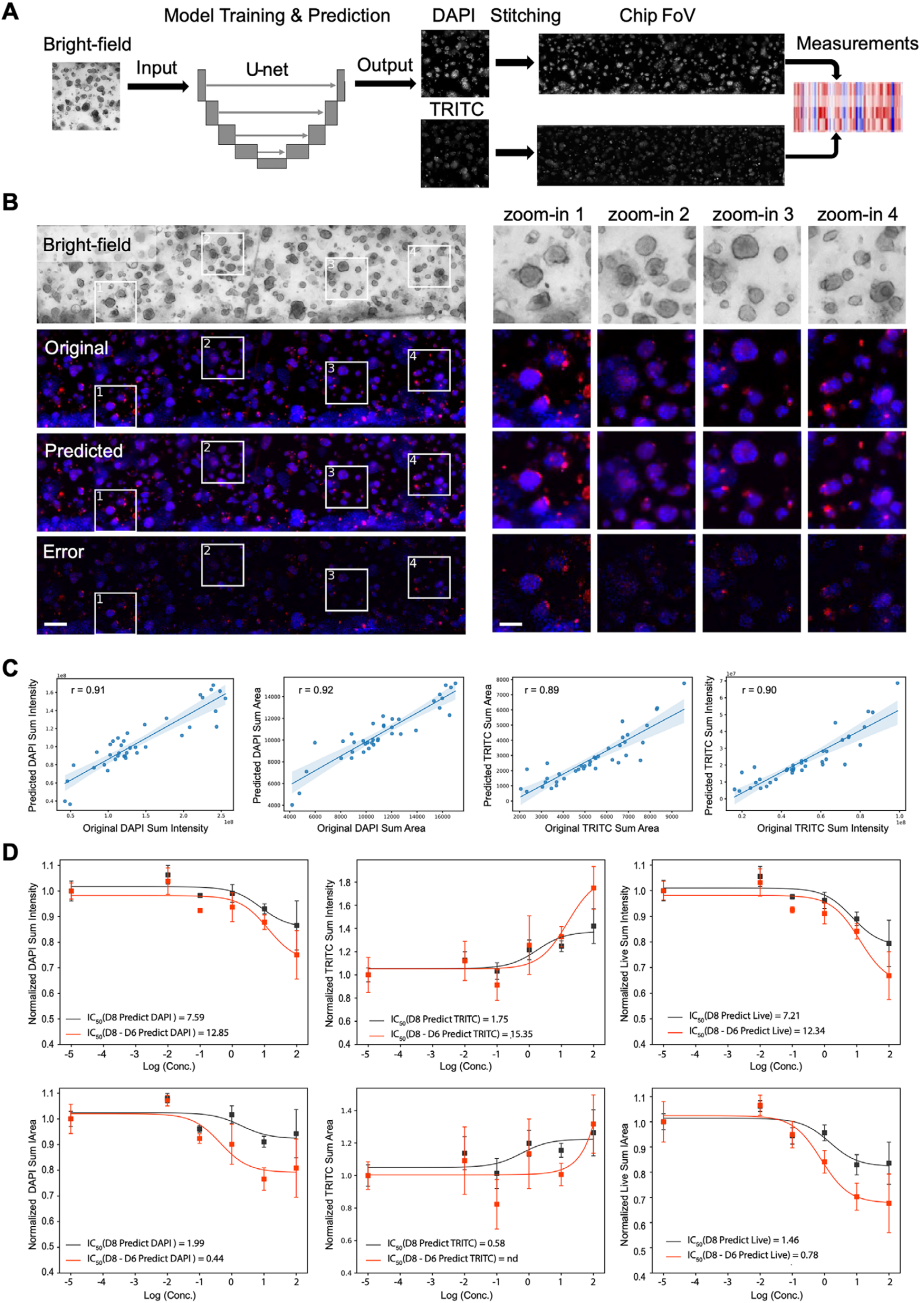
Deep learning‐enabled label‐free imaging analyses on‐chip to quantify drug effects. (A) Illustration of the analysis workflow. (B) Examples of original DAPI/TRITC images and the predicted DAPI/TRITC images from a BF image. The error maps and zoom‐in images showing correct prediction (zoom‐in 2&3) and misprediction (zoom‐in 1&4). Scale bars: 155 µm (whole chip) and 70 µm (zoomed‐in view). (C) Pearson correlations between experimental measurements and model predictions. The sum intensity/area ratios extracted from the original DAPI/TRITC images and model predicted DAPI/TRITC on the test dataset. (D) Dose‐response curve fittings and IC_50_ values based on sum intensity and sum area measurements. The black and red represent the average measurements from the DAPI channel (left), TRITC channel (middle) or live population (DAPI positive and TRITC negative, right) at the day‐8 endpoint, and day‐8 changes from the day‐6 baseline, respectively; n.d. means not determined. Data show mean ± s.d.; *n* = 4 chips.

To evaluate the feasibility of applying BF‐to‐fluorescence image mapping to our cancer‐on‐chip platform, we trained convolutional neural networks with a U‐net architecture [42]. The training dataset consisted of paired maximum‐intensity projected 2D BF and fluorescence images (4’,6‐diamidino‐2‐phenylindole (DAPI) and tetramethylrhodamine (TRITC) channels) of BxPC3 cells. Image pairs were acquired from the same imaging window, eliminating the need for image registration. Separate models were trained for each fluorescence channel, and their performance was assessed on an independent test set (Figure 5A). Model accuracy was quantified by comparing extracted features or measurements, such as sum intensity and sum area, from predicted and ground‐truth fluorescence images using the Pearson correlation coefficient (Figure 5B). The high correlation coefficients (0.89–0.92) demonstrated that model‐predicted fluorescence images reliably captured key features suitable for downstream analysis (Figure 5C). For reference, we also evaluated the test set using pixel‐wise metrics such as Structural Similarity Index Measure (SSIM) and Peak Signal‐to‐Noise Ratio (PSNR); however, these metrics were less biologically relevant and could not be directly used as an assessment for downstream analysis. The DAPI channel had an average SSIM of 0.6 and an average PSNR of 28, while the average SSIM and PSNR for the TRITC channel were 0.8 and 32, respectively. The lower SSIM and PSNR of the DAPI channel were primarily due to uneven staining in the test set, which was less severe in the TRITC channel.

Aggregated features at the chip level, including intensity and area from total (DAPI), dead (TRITC), or live (DAPI‐positive and TRITC‐negative), were used to assess drug responses by fitting dose‐response curves to chip‐level features across the concentrations. Traditionally, drug response evaluation relies on endpoint fluorescence images taken after treatment (e.g., Day 8 in this study). Using in silico staining, we generated fluorescence‐equivalent measurements at earlier time points, enabling more dynamic analysis. We compared three approaches: dose‐response curves based on endpoint fluorescence images alone (Day 8), changes from baseline DAPI measurements (Day 6 subtracted from Day 8), and changes from baseline live‐cell measurements (DAPI positive and TRITC negative). While all approaches detected gemcitabine dose responses, baseline‐normalized changes in live‐cell measurements demonstrated the highest sensitivity (Figure 5D).

In addition to enhanced sensitivity, in‐silico staining addressed experimental artifacts such as inhomogeneous staining (Figure S15A). Additionally, by enabling fluorescence‐equivalent image generation at multiple time points, this method facilitated longitudinal analyses that are not possible with traditional staining‐based assays. Longitudinal tracking provided insights into cellular growth and behavior before and after drug treatment, revealing dynamic factors that influenced treatment outcomes and were missed in endpoint analyses (Figure S15B,C). Overall, this deep learning‐based approach for label‐free imaging offers a scalable and high‐content tool for phenotypic analysis on cellular images from OC, offering spatial and temporal resolution of cell responses to drug treatment.

## 3 Discussion

In this work, we introduce OC‐Plex, a modular, scalable, and mass‐producible OC device suitable for high‐content imaging. Compared to existing OC platforms, our device presents multiple major advantages in design principle, manufacturability, and translational utility. While many existing systems rely on polydimethylsiloxane (PDMS), epoxy, or solvent‐based fabrication, OC‐Plex is made entirely from PS, a thermoplastic widely used in commercial cell culture labware due to its biocompatibility, optical clarity and excellent barrier properties, minimal absorption of small hydrophilic and hydrophobic molecules [43]. The combination of PS with injection molding and thermal bonding not only enables reproducible, industrial‐scale fabrication but also supports cost‐efficient production. To date, over 7000 devices have been produced and used across more than 10 independent or derivative studies, underscoring both scalability and robustness of this design for real‐world use.

While the fundamental geometry of the two‐channel microfluidic system may appear superficially similar to prior membrane‐free designs, OC‐Plex is structurally distinct. Instead of relying on microstructures, such as pillars, posts, or phase guides to confine hydrogels [14, 31, 44], our platform employs a trench‐based architecture that achieves hydrogel confinement through geometric expansion and contraction and surface functionalization (Figure S16). This barrier‐free interface between the tissue and perfusion channels eliminates the need for microstructures, reducing concerns related to drug absorption, autofluorescence, and fabrication costs. Moreover, this design supports unimpeded tissue‐tissue interactions between individual channels, a key advantage for modeling cancer cell invasion, migration, angiogenesis, metastasis to distant organs, and the interactions between cancer cells and immune cells within the TME [45]. Additionally, the modular design of the chip and reservoir layer offers the flexibility to convert the current single‐OC to multi‐OC configurations or other channel geometries with more than two channels or with a larger form factor, reinforcing adaptability and design flexibility.

PDOs have not been integrated into prospective clinical decision‐making, partially due to the lack of scalable, quantitative platforms for drug response profiling in clinical settings. By combining high‐throughput OC platforms and an image‐based phenotypic analysis pipeline, our platform has the potential to address these technical hurdles. To demonstrate the feasibility of our strategy, we performed drug sensitivity screening using both cell lines and patient‐derived cancer cells. The reduced drug sensitivity consistently observed across lung, pancreatic, and colon cell lines and patient‐derived cells corroborates previous publications and mirrors in vivo findings [46, 47]. Possible mechanisms underlying this insensitivity include reduced drug penetration under 3D conditions, altered expression of genes and proteins involved in drug‐resistance mechanisms (such as increased expression of drug efflux pumps or anti‐apoptotic pathways), and the 3D matrix itself acting as a barrier to drug diffusion [48]. However, our diffusion analysis (Figure S1) confirms that molecular equilibrium is reached within 1 h, suggesting minimal difference in initial drug availability between 2D and 3D on‐chip cultures. Furthermore, our results indicating that drug responses from day 1 treatment on‐chip approximate those from 2D cultures suggest that the matrix itself is unlikely to be the reason. Instead, increased proliferation over time in 3D culture may lower drug exposure per cell, potentially contributing to the elevated IC_50_ values seen at later time points. Future work is required to decipher the precise molecular mechanisms.

We also provide multiple proof‐of‐concept demonstrations of OC‐Plex in functional precision oncology. In our cancer chip model, pemetrexed was not effective against patient‐derived squamous cell carcinoma regardless of the treatment durations (3 or 6 days), despite strong efficacy in traditional 2D testing. This result aligns with clinical recommendations for pemetrexed use in patients with NSCLC, such as adenocarcinoma and large cell carcinoma, but not for patients with squamous cell carcinoma [49, 50]. These results highlight the capacity of our platform to better recapitulate clinically relevant, patient‐specific drug responses than conventional 2D systems. In addition, the platform supports immune–tumor co‐culture and longitudinal functional assessment of immune checkpoint blockade. Using pre‐activated PBMCs co‐cultured with patient‐derived gastric cancer organoids, treatment with the PD‐1 inhibitor sintilimab elicited dose‐ and time‐dependent antitumor activity. Although limited in scope, this experiment illustrates the extensibility of the OC‐Plex platform toward modeling immune‐mediated therapeutic responses that are difficult to assess using conventional organoid assays. Together, these findings underscore the potential of our OC‐Plex platform to model complex therapeutic mechanisms and support personalized treatment decisions, although future work involving a larger cohort of patients in a prospective trial is needed to demonstrate its clinical validity. Indeed, several studies are underway using the OC‐Plex platform to further evaluate the concordance between patient‐specific‐OC‐predicted drug sensitivity and patient outcomes in CRC and other solid tumors.

While ATP‐based assays have been widely used in functional precision medicine, our comparative studies suggest that imaging presents a superior readout. Imaging offers several advantages in functional precision oncology, including low cost, the ability for multiplexing, providing spatial information, and supporting label‐free, kinetic analysis [51]. BF imaging excels in tracking dynamic cellular processes over time, facilitating longitudinal studies, whereas fluorescence imaging is limited to providing snapshots of cells at specific instances. Recently, deep learning approaches have been successfully applied to predict fluorescent markers from BF images, known as in silico labeling/staining [24, 51]. Here, we demonstrate, for the first time, the integration of a deep learning‐based BF‐to‐fluorescence image translation method for cancer OCs, uniquely facilitating longitudinal monitoring of cell growth and dynamic behaviors without fluorescence labeling. Our approach of using a deep learning model to achieve BF‐to‐fluorescence image translation streamlines the imaging workflow, enhances efficiency, and combines the advantages of both imaging modalities. In silico labeling/staining can replace the time‐consuming sample preparation process with computational techniques and enable longitudinal analyses of predicted fluorescence images. Unlike conventional single‐timepoint analyses, our innovative approach enables precise measurement of baseline changes (i.e., pre‐ and post‐drug treatment), dramatically enhancing sensitivity to drug dose responses. This method, unattainable through traditional empirical staining and endpoint analyses, also effectively compensates for missing or inconsistent signals arising from uneven staining—an inherent and common challenge in 3D cell culture environments. Moreover, the deep learning architecture employed in our platform is highly efficient, demonstrating ease of training and rapid inference speeds. These features make the proposed in silico staining method practically deployable at scale, particularly beneficial in our advanced, high‐throughput organ chip platforms where stable, fast, and accurate predictions are essential for real‐world applications in cancer precision medicine.

In summary, OC‐Plex bridges engineering, biology and AI analytics to overcome key limitations in translating functional oncology into clinical practice. Its unique trench‐based geometry and thermoplastic construction offer a highly scalable and cost‐effective solution compatible with industrial manufacturing and automated workflows. Coupled with deep learning‐based image analyses, the platform enables high‐content, quantitative, and longitudinal assessments of tumor responses. Although further work incorporating broader tumor microenvironment components and larger patient cohorts are warranted to further validate predictive accuracy in clinical trials, OC‐Plex represents a robust and practical foundation for high‐throughput, precision‐guided cancer therapy.

## 4 Materials and Methods

### 4.1 Chip Design and Fabrication

The microfluidic channels, OC‐Plex 8, and OC‐Plex 32 assembly were designed in SOLIDWORKS (Dassault Systems). The injection molding of the OC‐Plex was carried out at a third‐party manufacturer where a metal injection molding tool was machined with the inverse of the original design. Compared to everyday injection molded plastics, sub‐micron surface roughness, micron‐level deformation, and <1° wall‐draft may change the functionalities of the design geometries; furthermore, achieving micron level flatness is critical in enabling high content imaging. A rigorous design for manufacturing (DFM) process and mold flow (MF) analysis was carried out with the manufacturer to ensure success in injection molding. The molded parts are cleaned with 99.92 propanol (Sigma–Aldrich, 34863) and thermal bonded to a piece of die‐cut 200‐µm‐thick PS film. Due to the strict flatness requirement, a proprietary fixturing and bonding strategy was developed to achieve a bond that was robust at elevated temperatures and humidity while maintaining the geometric dimensions specified during injection molding. The bonded part was subsequently functionalized with polyethylene glycol (PEG) and sterilized with UV as previously described [52] to create sterile hydrophilic microchannels. The functional devices were packaged and ready for use.

### 4.2 Cell Culture

BxPC‐3 cells (RRID:CVCL_0186, ATCC, CRL‐1687) were cultured in T75 flasks in RPMI‐1640 (Gibco, 11835030) supplemented with 10% fetal bovine serum (Gibco, A3160502), 100 U/mL of penicillin, and 100 µg/mL of streptomycin (Gibco, 15140122) at 37°C in 5% CO2. A549/GFP cells (RRID:CVCL_JY87, Cell Biolabs, AKR‐209) were cultured in T75 flasks with DMEM, high glucose (Gibco, 11965092). Medium was supplemented with 10% fetal bovine serum (Gibco, A3160502), 0.1‐mm MEM Non‐Essential Amino Acids (Gibco, 11140050), 1% penicillin‐streptomycin (Gibco, 15140122) at 37°C in 5% CO_2_. All cell culture was performed in a humidified incubator at 37°C in 5% CO_2_. HCT116 (RRID:CVCL_0291, CCTCC, SCSP‐5076) and HT‐29 cells (RRID: CVCL_0320, CCTCC, SCSP‐5032) were cultured in T75 flasks with McCoy's 5A medium modified (Gibco, 12330031), supplemented with 10% fetal bovine serum (Gibco, A3160502), 100 U/mL of penicillin, and 100 µg/mL of streptomycin (Gibco, 15140122) at 37°C in 5% CO_2_. PBMCs were cultured in T75 flasks with Lymphocyte Medium (Lonza, BEBP02‑054Q), supplemented with 10% fetal bovine serum (Gibco, A3160502), 100 U/mL of penicillin, 100 µg/mL of streptomycin (Gibco, 15140122), 3000 IU of recombinant human interleukin‐2 (IL‐2), and 25 µL/mL of ImmunoCult Human CD3/CD28 T Cell Activator (Stemcell, #10971) at 37°C in 5% CO_2_. All cell lines were tested free of microbial contamination.

The primary lung cancer cells used in this study were isolated from the primary tumor tissues of lung squamous cell carcinoma (LUSC) patients undergoing surgical treatment. The use of discarded tumor tissues complies with all relevant ethical regulations and has been approved by the Institutional Review Board of Henan Academy of Innovations in Medical Science. Briefly, fresh tumor tissues were isolated and promptly transferred to 10‐cm dishes. The samples underwent 2–3 washes with PBS and were then finely sliced into 0.1–1‐mm fragment using a scalpel. Subsequently, cells were dissociated utilizing the Tumor Dissociation Kit, human (Miltenyi, 130‐095‐929). The isolated cells were seeded into T25 flasks containing DMEM, high glucose (Gibco, 11965092) supplemented with 5% fetal bovine serum (Gibco, A3160502), 10 ng/mL of bFGF (PeproTech, 100–18B), 10 ng/mL of EGF (PeproTech, AF‐100‐15), and 10 ng/mL of IGF (PeproTech, 100–11). The culture medium was refreshed twice weekly until a confluent monolayer was achieved.

For gastric cancer organoid culture, 50 µL of the gel–cell mixture was aliquoted into each well of a 24‑well plate. The plate was then placed in a 37°C, 5% CO_2_ incubator for 30 min to allow gel polymerization. Subsequently, 800 µL of Human Gastric Cancer Organoid Medium (Mogengel, MA‑0807T008LP) was added to each well. The medium was replaced every 2–3 days.

### 4.3 Cell Culture in OC‐Plex

The OC‐Plex was used for microfluidic cell culture. Rat tail col‐1 (R&D Systems, 3447‐020‐01) prepared at 4 mg/mL with a 1:1:8 ratio of 3.7% (w/v) sodium bicarbonate with a pH of 9.5 to 1‐m HEPES to rat tail col‐1, was used as a hydrogel matrix for BxPC3 cell culture and Cultrex Reduced Growth Factor BME (R&D Systems, BME001‐05) were used as the hydrogel matrix for A549, HCT116, HT‐29, and primary lung cancer cell cultures. Cells were dissociated, pelleted, and resuspended in hydrogel matrix. Hydrogel/cell suspension was kept on ice. Using an electronic single channel pipette, 1.5 µL of hydrogel/cell suspension was seeded into the gel channel. The device was placed in a petri dish with a clean room cloth with 10 mL of molecular‐grade biology water and 50 µL of PBS above the ribbed area to prevent gel deformation. Devices were incubated at 37°C, 5% CO_2_ for 30 min (for BME) or 60 min (for col‐1) to allow polymerization. After incubation, 60 µL of medium was added to the perfusion channel and 20 µL of medium to atop of each gel port to each OC‐Plex chip for a total volume of 100 µL per chip. The OC‐Plex devices were placed in a humidified incubator, and the medium was replaced every 2–3 days.

### 4.4 Drug Treatment

The compounds used in these studies, gefitinib (Medchem Express, HY‐50895), gemcitabine (MedChem Express, HY‐17026), osimertinib (HY‐15722), and savolitinib (HY‐15959), paclitaxel (HY‐B0015), regorafenib (HY‐10331), and pemetrexed (HY‐10820) were individually prepared in sterile DMSO as 1000x stocks (0.01, 0.1, 1, 10, 100 mm), aliquoted, and stored at −80°C. On the day of dosing, each compound was diluted with growth medium at a ratio of 1:1000 to achieve final concentrations of 0.01, 0.1, 1, 10, and 100 µm. Vehicle control was prepared by diluting 100% sterile DMSO to 0.1%. For cisplatin (HY‐17394) and oxaliplatin (HY‐17371), the drugs were prepared as 2 mm master mixes in sterile water and diluted in growth medium to final concentrations of 0.01, 0.1, 1, 10, 100, and 200 µm, respectively, before use. Medium was removed completely from each OC‐Plex chip or 96‐well and replaced with medium containing compound or vehicle for 48 h prior to endpoint viability assessment. For the drug treatment of human gastric cancer organoids, sintilimab was prepared as a 10‐mg/mL stock solution in sterile PBS, aliquoted, and stored at −80°C. On the day of treatment, the stock was diluted in culture medium to obtain working concentrations of 10 µg/mL (1:50 dilution) and 200 µg/mL (1:1000 dilution).

### 4.5 Human Gastric Cancer Organoid Co‐Culture Assay

To further evaluate the drug testing capability of the OC‑Plex 8 microfluidic device, human gastric cancer organoids were dissociated into single cells or small clusters and quantified with an automated cell counter (Invitrogen Countess 3). PBMCs from healthy donors (SAILYBIO, XW0809051) were isolated and activated using ImmunoCult Human CD3/CD28 T Cell Activator (Stemcell, #10971). For live‑cell tracking, PBMCs were stained with 25 µm of lipophilic fluorescent dye DiO (Beyotime, C1038) by incubation at 37°C for 25 min, with gentle resuspension every 5 min to ensure uniform labeling. The labeled PBMCs were then mixed with organoid cells at an effector‑to‑target ratio of 5:1 (PBMCs: 1 × 10^7^ mL^−1^; organoid cells: 2 × 10^6^ mL^−1^) in Matrigel (Corning, #354230). 1.5‐µL aliquot of the cell–Matrigel mixture was loaded into the gel channel of the microfluidic device. After incubation at 37°C with 5% CO_2_ for 60 min to allow gel polymerization, 100 µL of culture medium with 1:1 mixture of gastric cancer organoid medium (Mogengel, #MA‑0807T008LP) and PBMC medium (LONZA, #BEBP02‑054Q) was added to both the gel and perfusion channel ports. The medium was replenished every 2–3 days until drug treatment. Following 5 days of co‑culture, the cultures were treated with either vehicle control (blank control), 10 µg/mL of sintilimab (MCE, HY‑P99048), or 200 µg/mL of sintilimab. Bright‑field and fluorescence images were acquired at 0, 24, and 48 h post‑treatment to monitor morphological changes and cell viability values. At the 48‑h endpoint, cells were stained with Hoechst 33342 (Thermo Scientific, 62249) and PI (Aladdin, P113815) for final viability assessment.

### 4.6 Human Materials

All experiments involving human materials were conducted in accordance with institutional and ethical guidelines and were approved by the appropriate ethics committees. Ethical approval was obtained under reference numbers 1514 (2023) (West China Hospital of Sichuan University) for the lung cancer study and 2021‐437‐005 (Henan Cancer Hospital) for the gastric cancer study. Written informed consent was obtained from all human tissue donors prior to sample collection. All donor information was anonymized during data analysis to ensure participant confidentiality.

### 4.7 Viability Staining

Viability staining solution was prepared by diluting Hoechst 33342 by 1:2000 to achieve a final concentration of 10 µm, EthD‐2 (Invitrogen, E3599) by 1:1000 to achieve 1 µm, PI by 1:125 to 80 µm, and optionally, CellEvent Caspase‐3/7 green detection reagent (Invitrogen, C10423) by 1:500 to achieve 4 µm in complete growth medium. Prior to staining, the medium was removed completely from OC‐Plex and 96‐well plate and replaced with staining solution. Stain was incubated for 2 h for OC‐Plex and 40 min for a 96‐well plate in a humidified incubator (37°C, 5% CO_2_) before imaging. For OC‐Plex, staining solution was mixed by pipetting up and down in the perfusion channel and ECM ports after 1 h to enhance staining efficiency.

### 4.8 Automation

Automation was performed with Integra Assist Plus (Integra, 4505). Automated handling steps include hydrogel loading, media exchange and drug dosing. An integra D‐One 12.5 µL single channel pipette (Integra, 4531) was used to repeat dispense hydrogel into OC‐Plex chips. An 8‐channel integra Voyager pipette (Integra, 4723) was employed to dispense and aspirate cell culture media or drug dosing solutions for media exchange or drug treatment.

### 4.9 Image Acquisition and Processing

Imaging was conducted with an Agilent BioTek Lionheart LX Automated Fluorescent Microscope through a 4x air objective (Olympus Plan Fluorite 0.13NA #1220519). Automated imaging and processing protocols were established using BioTek Gen5 Software (Agilent). Each chip was acquired with two 10% overlapping fields of view and 19 Z‐slices (14‐µm step size) to encompass the entire gel channel height. Longitudinal imaging for cell growth monitoring was performed in the bright‐field channel. Pre‐analytical processing steps were applied to the bright‐field images, including stitching, 2D projection, digital phase contrast, kinetic frame registration, background subtraction, and smoothing. Live/Dead Imaging was acquired in DAPI channel (Excitation 377/50 nm, Emission 447/60 nm) for Hoechst 33342, TRITC channel (excitation 556/20 nm, emission 600/37 nm) for EthD‐2 or PI, and green fluorescent protein (GFP) channel (excitation 469/35 nm, emission 525/39 nm) for caspase‐3/7 or DIO. Fluorescence images for each channel underwent stitching, background subtraction and 2D projection before image analysis.

### 4.10 Image Analyses

Image analyses were performed using the BioTek Gen5 Software (Agilent). Processed bright‐field images were utilized for cell/aggregate‐segmentation to monitor cell growth over time. Each individual cell or cluster of cells was segmented as an object based on its intensity. The changes in mean object area over time of each OC‐Plex chip were quantified to represent the average growth of individual cells or clusters. Additionally, the changes in the sum area of all objects in each chip over time were calculated to indicate overall the cell growth the chip‐level.

For viability imaging assay, the image‐analysis strategy involved confining ROI and feature‐extraction using a fluorescence intensity‐based masking process. Individual cells or clusters of cells were segmented as objects by applying a mask using thresholding method based on Hoechst 33342 intensity. Within the object mask, dead cells and apoptotic cells were identified based on EthD‐2 intensity and caspase‐3/7 intensity, respectively. Each object was further assessed for a variety of intensity and morphology related metrics. Following the calculation of object morphology and intensity, objects were categorized into “Single‐Cell” and “Aggregate” groups based on their sizes. The viable area was quantified by deducting the EthD‐2 mask area from Hoechst33342 mask, and the viability% was quantified by using viable area normalized to the sum area of all cells.

### 4.11 Z’‐Factor Analyses

Z’‐factor was calculated based on the equation provided below:

$$Z^{\prime }=1-3\times \left(\sigma c^{+}+\sigma c^{-}\right)/\left|\mu c^{+}-\mu c^{-}\right|$$

where σc+ and σc‐ represent the standard deviations of the positive control and negative control respectively and μc+ and μc‐ represent the means of the positive control and negative control respectively [35]. For drug sensitivity testing, the vehicle control (0.1% DMSO) served as the negative control; 100 µm osimertinib and 100 µm gemcitabine served as the positive controls for BxPC‐3 and A549 models, respectively. Z’‐factor was calculated using all imaging metrics and CTG.

### 4.12 Fluorescent Tracer Penetration Assay

Col‐1 gel was used to access the diffusion rate from the perfusion channel to the gel channel. Rhodamine B was used as the tracer molecule for its hydrophilicity and fluorescence. A 200 µm of rhodamine B (Sigma–Aldrich, 83689‐1G) solution was prepared in 1x PBS and loaded into the perfusion channel. The OC‐Plex chip with the gel and rhodamine B was allowed to incubate at room temperature and imaged with bright‐field at time 0, 2.5, 5, and 10 min. To increase fidelity, kinetic imaging was carried out to assess diffusion rate with 1 and 10 µm of rhodamine B and Cy5‐Dextran. A 70‐kDa Cy5‐Dextran (Sigma–Aldrich, 90718‐1G) was added to the experiment to account for a large hydrophilic molecule. The OC‐Plex chips were incubated at room temperature for 1 h and 2 h for 1‐µm solution and 10‐µm solution, respectively. During the incubation period, fluorescent images were taken every 2 min with an Agilent BioTek Lionheart LX Automated Fluorescent Microscope.

### 4.13 Cell Viability Assay

To quantify cell viability in the OC‐Plex device, culture medium was removed from both channels. Subsequently, 100 µL of CellTiter‐Glo 3D Reagent (Promega, G9681) was aspirated and dispensed into the open medium channel and ECM ports. This process was repeated for all chips in the device. Following this, the device was placed on an orbital shaker at room temperature for 1 h. Concurrently, a rATP standard (Promega, P1132) was prepared by diluting the 10‐mm stock 1:1000 in culture medium. 50 µL of this medium was added to wells A2‐A12 of a white opaque 96‐well plate, and 100 µL of the diluted rATP standard was placed into well A1. This standard was serially diluted by transferring 50 µL from well A1 to well A2 and so on, mixing each well 5–7 times, with well A12 reserved as the blank. After shaking the plate for 5 min, it was incubated for 25 min. Upon completion of the 1‐h incubation of the devices, the CellTiter‐Glo 3D Reagent was aspirated from the devices and transferred to the 96‐well plate, with each chip's reagent in separate wells. Luminescence was read immediately with an integration time of 0.25–1 second per well.

### 4.14 Model Training, Validation, and Testing

We employed the U‐net convolutional neural network due to its demonstrated performance in image‐related tasks. Moreover, the choice and development of the U‐net‐based algorithm took into account reasonable training time as well as fast and stable inference in a production environment for OC devices at scale rather than in a research setting. The deep learning model was trained and validated on a BxPC3 cell line dataset of a total of 756 image pairs for each channel (BF and fluorescence images were acquired from the same imaging window, so no registration was required). The images for training were carefully screened by human experts to ensure that only those without staining quality issues or artifacts were included. We split the dataset into training, validation, and test sets with a ratio of 7:2:1. The validation set was used for hyperparameter tuning and the test set was used to evaluate the final model performance.

The Z‐projection image has a region of interest of ∼1000 by ∼300 pixels and was cropped into patches of 224 by 224 pixels with overlapping regions as the input image to the U‐net model. We used a batch size of 8 and the Adam optimizer with a learning rate set to 10^−4^ for weight updates during the optimization process. The model was trained using a mean squared error (MSE) loss over 150 epochs. The model performance was evaluated via Pearson's correlation coefficient on extracted features or measurements between predicted fluorescence images and ground‐truth images.

A single Nvidia T4 GPU was used for training, which took about 1 h. The inference time for one chip (∼1000 by 300 pixels per image) was approximately 0.1 s.

### 4.15 Image Feature Extraction and Analyses

We extracted features from the predicted chip‐level images after stitching image crops. The post‐processing steps include Gaussian smoothing, artifact removal, thresholding (Two‐class Otsu thresholding used for DAPI and Three‐class Otsu used for TRITC), and threshold‐based segmentation. The sum intensity and sum area are the two most representative measurements of the chip‐level properties. We also measured cell viability using different metrics. The live area/intensity in Figure 2 is defined as the DAPI area/intensity minus the TRITC positive area/intensity within the DAPI mask. The area/intensity‐based viability in Figure 3 is the ratio of live area/intensity to DAPI area/intensity.

The longitudinal predictions utilized the relationship between BF and fluorescence images learned at the endpoint images (Day 8) and applied to BF images taken at Days 0, 2, 4, 6. The image features at earlier timepoints were extracted in the same manner as the endpoint. Day 8 measurements subtracted by Day 6 measurements represent the changes from the baseline due to drug treatment, which alleviate the impact of chip‐to‐chip variation. The earlier timepoints track the cell behavior before drug treatment.

### 4.16 Statistical Analyses

All experiments were repeated at least two times. Data are displayed as mean values ± standard deviations (s.d.) unless otherwise noted. Graphing and statistical comparison of the data were performed using GraphPad Prism 9.0. Two‐group comparisons were assessed using the two‐tailed Student's *t* test; comparison of three or more groups were analyzed by one‐way ANOVA with Tukey's multiple comparisons test. *p* values <0.05 were considered statistically significant; ^*^ *p* < 0.05; ^**^ *p* < 0.01; ^***^<0.001; n.s., not significant.

## Author Contributions

L.S., Y.S.Z., X.X., and H.B. conceptualized the study and guided experimental designs. YC.Y., B.X., J.MC., XH.H., J.M., and H.B. drafted the manuscript with input from all authors. YC.Y., B.X., and J.MC. performed experiments involving pancreatic cancer, colon cancer, and A549 cells, respectively. B.X. performed experiments involving patient‐derived lung cancer. XH. H. conducted an engineering test of the device. J.M. and Z.X. performed deep‐learning based image analysis. All other authors contributed to the revision of the manuscript. The final version of the manuscript has been approved by all authors.

## Conflicts of Interest

Competing interests: YC.Y., J.MC., XH.H., J.M., T.M., G.K., J.S., H.S., A.F., P.G., X.Q., Z.X., X.X., H.B. are currently employed with and own equity in Xellar Inc., a company that developed 3D Organ Chip Culture for drug discovery. P.G. and Y.S.Z. serve on the scientific advisory board of Xellar Inc. and own equity. Y.S.Z. received a sponsored research agreement from Xellar Inc. on a related topic approved by the Brigham and Women's Hospital.

## Supporting information




**Supporting File 1**: advs74140‐sup‐0001‐SuppMat.pdf.

**Supporting File 2**: advs74140‐sup‐0002‐Video S1.mp4.

## Data Availability

The data that support the findings of this study are available from the corresponding author upon reasonable request.
