# Supplementary material for: Deep Learning‐Powered Scalable Cancer Organ Chip for Cancer Precision Medicine
Source: Adv Sci (Weinh). 2026 Feb 3;13(26):e16660. doi: 10.1002/advs.202516660 (PMC13159154; doi:10.1002/advs.202516660)
Supplement: Supplementary file 1 — Supporting File 1: advs74140‐sup‐0001‐SuppMat.pdf. [file ADVS-13-e16660-s001.pdf]

Supplementary Materials for

Deep Learning-Powered Scalable Cancer Organ Chip for Cancer Precision Medicine

Yu-Chieh Yuan[1#], Beibei Xu[2#], Jenna McCormack[1#], XuHai Huang[1#], Jingzhe Ma[1#], Thomas Marshall[1], Yacong Sun[2], Hardeep Singh[1], Alyssa Fanelli[1], Gauri Kulkarni[1], Ji Hye Seo[1], Paige Gilbride[1], Bing Wei[3,4], Bo Wang[3,4], Yanyan Liu[5], Fei Ma[6], Lin Zhou[1,8], Shuyang Wang[7], Xiaohua Qian[1,8], Zhiyong Xie[1,8], Polina Golland[9], Longlong Si[2,10*], Yu Shrike Zhang[11*], Xin Xie[1,4,8*], Haiqing Bai[1,8*]

[1]Xellar Biosystems, Boston, MA, 02129, USA

[2]CAS Key Laboratory of Quantitative Engineering Biology, Shenzhen Institute of Synthetic Biology, Shenzhen Institute of Advanced Technology, Chinese Academy of Sciences, Shenzhen, China

[3]Department of Molecular Pathology, Henan Cancer Hospital, Zhengzhou, Henan 450000, China

[4]Henan Key Laboratory of Molecular Pathology, Zhengzhou, Henan 450000, China

[5]Department of Internal Medicine, Henan Cancer Hospital, Zhengzhou, Henan 450000, China

[6]Department of General Surgery, Henan Cancer Hospital, Zhengzhou, Henan 450000, China

[7]Department of Pathology, School of Basic Medical Sciences, Fudan University, Shanghai 200433, China

[8]Henan Academy of Innovations in Medical Science, Zhengzhou, Henan 450000, China

[9]Computer Science and Artificial Intelligence Laboratory, Massachusetts Institute of Technology, Cambridge, MA 02142, USA

[10]University of Chinese Academy of Sciences, Beijing 100049, China

[11]Division of Engineering in Medicine, Department of Medicine, Brigham and Women's Hospital, Harvard Medical School, Cambridge, MA 02142, USA

[#]These authors contribute equally to this work.

[*]Corresponding authors: Longlong Si, ll.si@siat.ac.cn; Yu Shrike Zhang, yszhang@bwh.harvard.edu; Xin Xie, xxie@xellarbio.com; Haiqing Bai, hbai@xellarbio.com

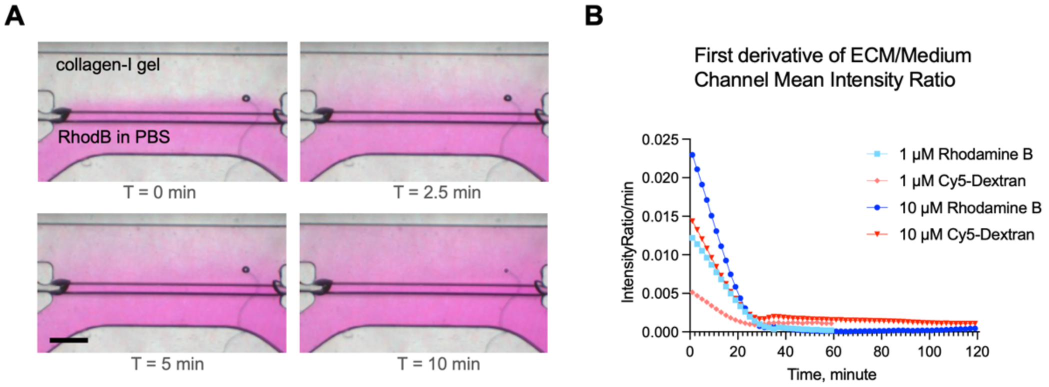

Supplementary Figure 1. Permeability assay on Chip. (**A**) Representative images showing the diffusion of rhodamine B from the perfusion channel into the gel channel. Scale bar: 500 µm. (**B**) Quantifications of the diffusion rates.

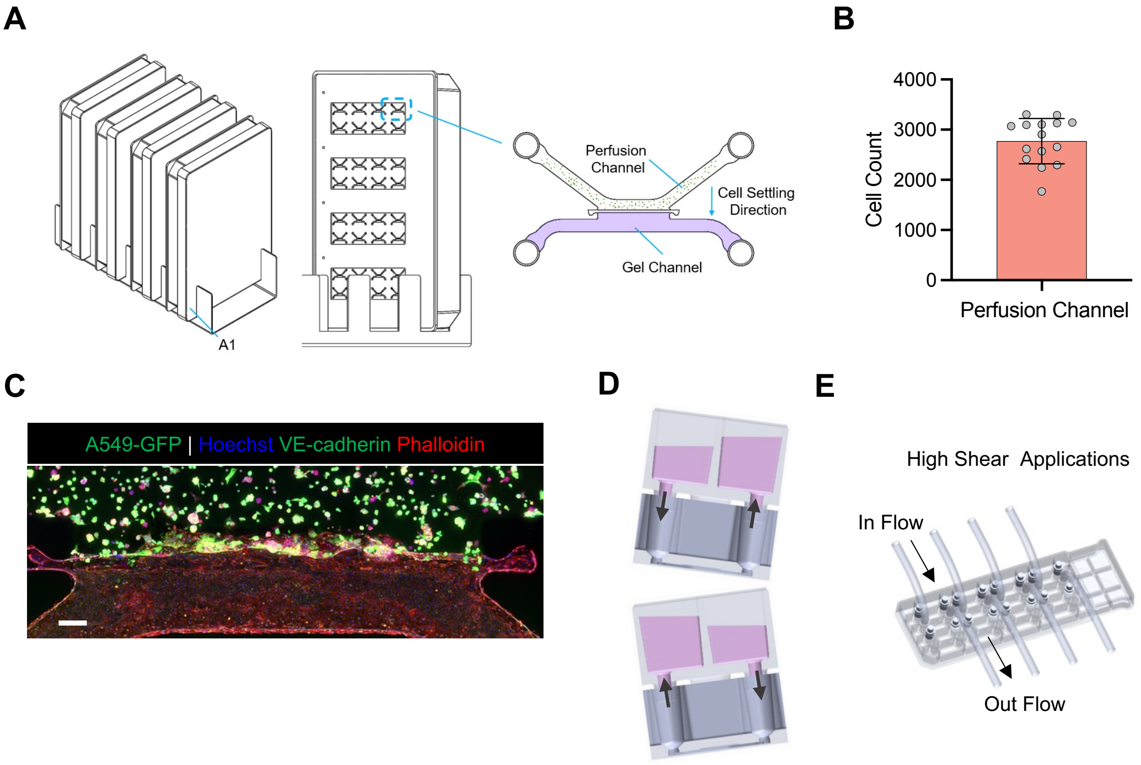

Supplementary Figure 2. Support of coculture on-chip. (**A**) Devices placed in a stainless-steel carrier to allow attachment of cells on the gel interface. (**B**) Image-analysis results showing consistent cell counts, each dot represents one chip. Data show mean ± s.d; n=15. (**C**) Immunostaining showing the coculture of A549-GFP cells in the gel channel and HUVECs in the perfusion channel. Cells were stained with VE-cadherin (green) and Phalloidin (red) at 9 days after coculture. Scale bar: 100 µm. (**D**) Dynamic culture with reservoir and shaker to allow flow. (**E**) Chips connected via tubing to peristaltic pump for high shear applications.

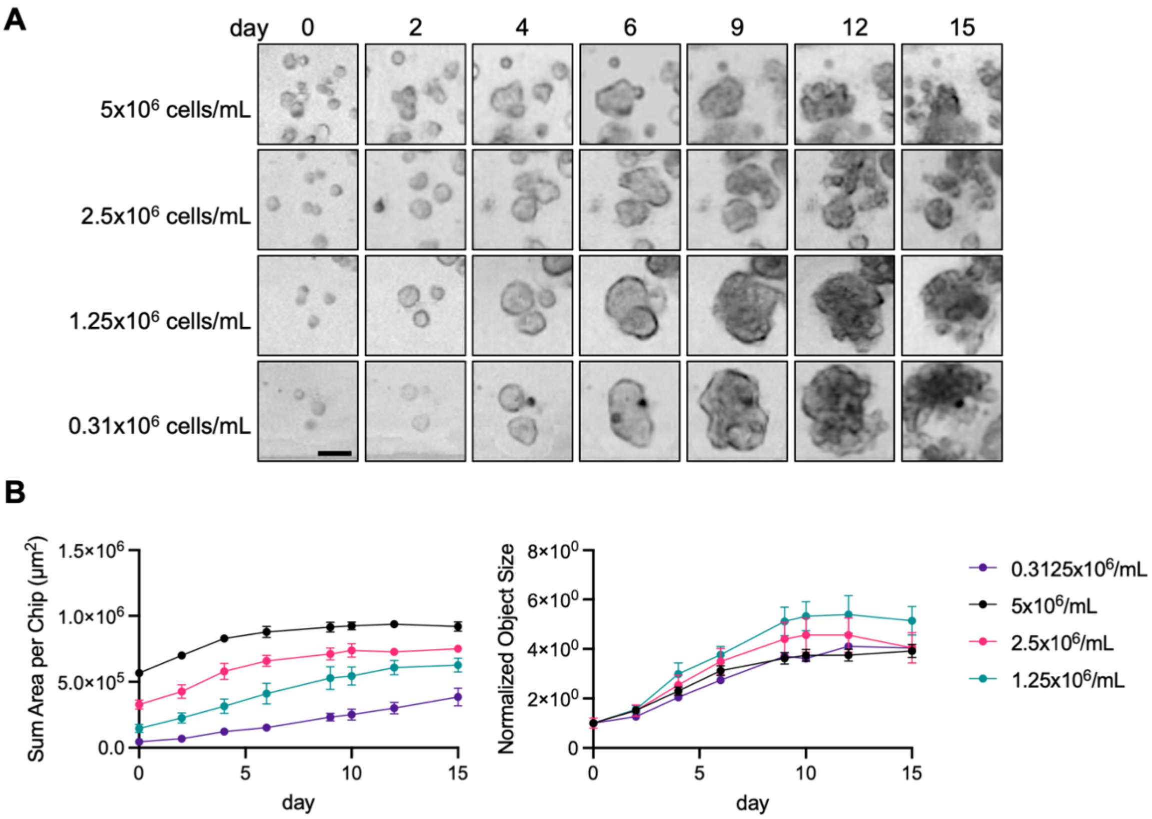

Supplementary Figure 3. Growth of pancreatic cell line on-chip. (**A**) Representative BF images of single-cell-embedded BxPC-3 cells growth at four seeding densities ranging from 0.31×10^6-5×10^6 cells/mL over 15–18 days of culture period. Scale bar: 50 µm. (**B**) Longitudinal BF image-analysis results of BxPC-3 growth at different densities on OC-Plex32 chips showing sum area per chip and normalized object size. Values were normalized to day 0. Data show mean ± s.d.; n=4 chips.

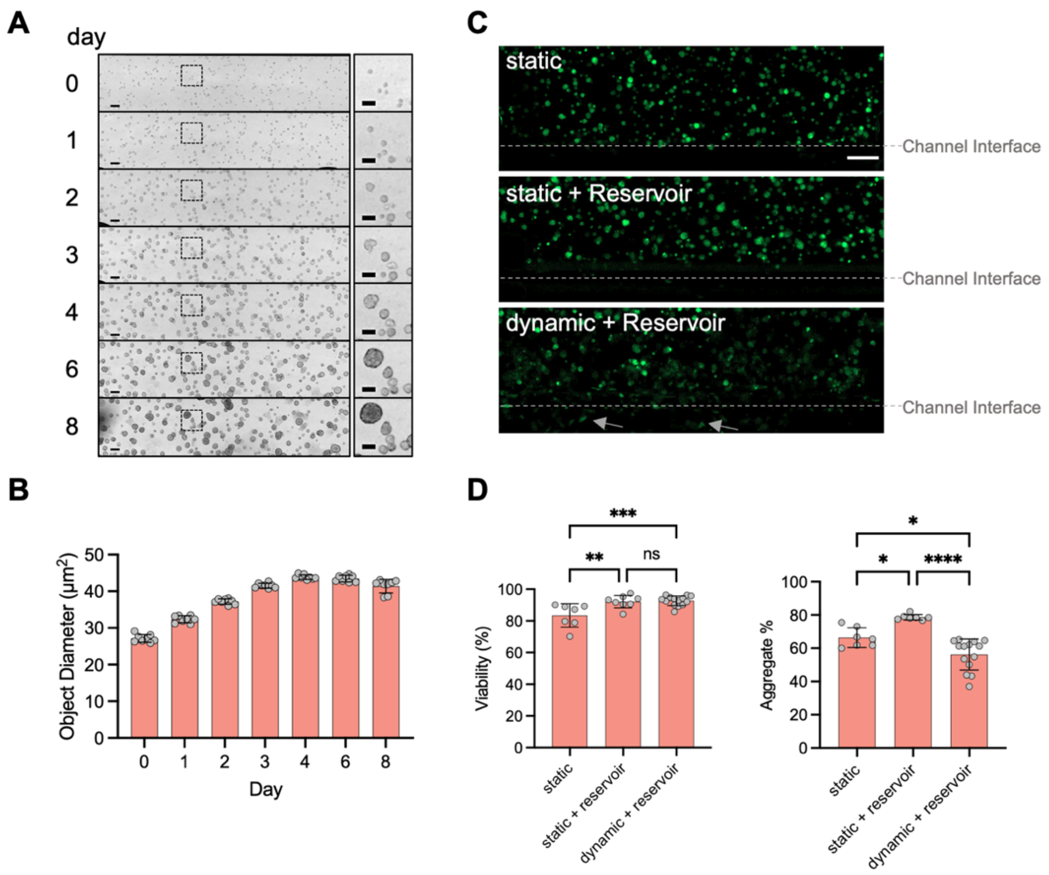

Supplementary Figure 4. Growth of A549 lung cell line on-chip. (A) Representative brightfield images showing single-cell embedded A549 growing for 8 days in the same OC-Plex chip cultured statically. Scale bar: 100 µm (whole chip) and 50 µm (zoomed-in view). **(B)** Longitudinal brightfield image analysis results for A549 growth in OC-Plex showing the mean object diameter from day 0 to day 8. Data show mean ± s.d.; n=8 chips. (**C**) Representative chip images showing A549-GFP cells at day 7 of culture in static with reservoir or without reservoirs and dynamic culture using a shaker set a 7-degree tilt angle and a hold-time of 8 minutes, corresponding to flow rate of 2.7 µL/min. Arrows indicate cells migrating from the gel channel to the perfusion channel. Scale bar: 200 µm. (**D**) Image analysis results of viability (left) and percentage of aggregates (right). Data show mean ± s.d.; n=7–14 chips; one-way ANOVA with Turkey's multiple comparisons test.

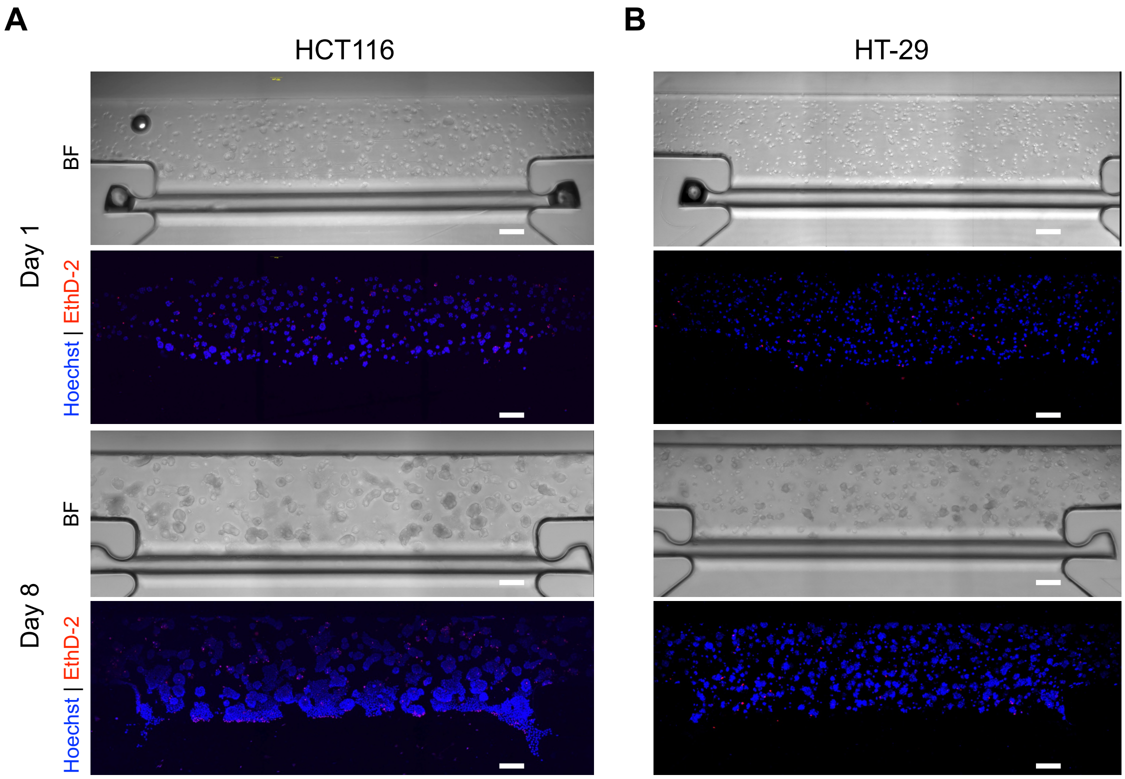

Supplementary Figure 5. Growth of colon cell line on-chip. Representative images of HCT116 (**A**) and HT-29 cells (**B**) cultured on the OC-Plex device for 1 day to 8 days, respectively. BF indicates bright-field image, and Hoechst/EthD-2 indicates a representative image of viability assessment with Hoechst33342 and EthD-2 staining. Scale bars: 50 μm.

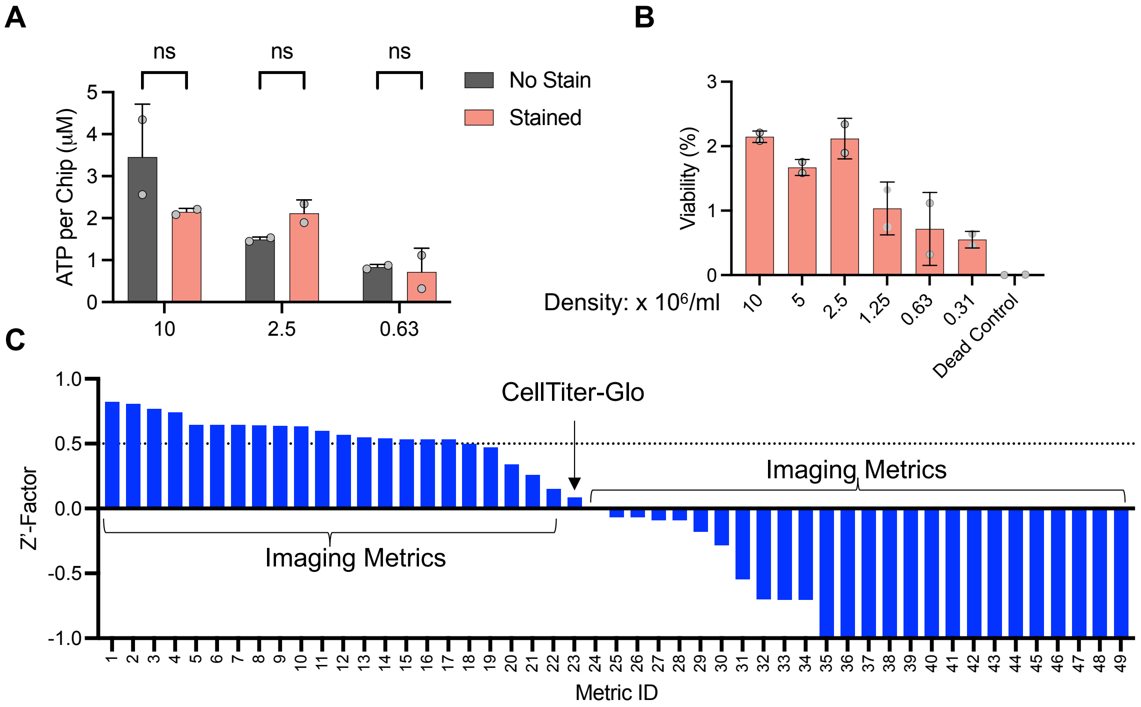

Supplementary Figure 6. Effect of live/dead staining on ATP assay. (**A**) Comparison of the CellTiter-Glo ATP results with and without Hoechst 33342 and EthD-2 staining addition. Data show mean ± s.d.; N=2 chips. (**B**) ATP results obtained from chips stained with Hoechst 33342 and EthD-2. The chips were seeded at densities ranging from 0.31×10^6 to 5×10^6 cells/mL and imaged for viability prior to the ATP assay. Data show mean ± s.d.; n=2 chips. (**C**) Histogram showing the Z'-factor values of all imaging metrics and the CellTiter-Glo assay.

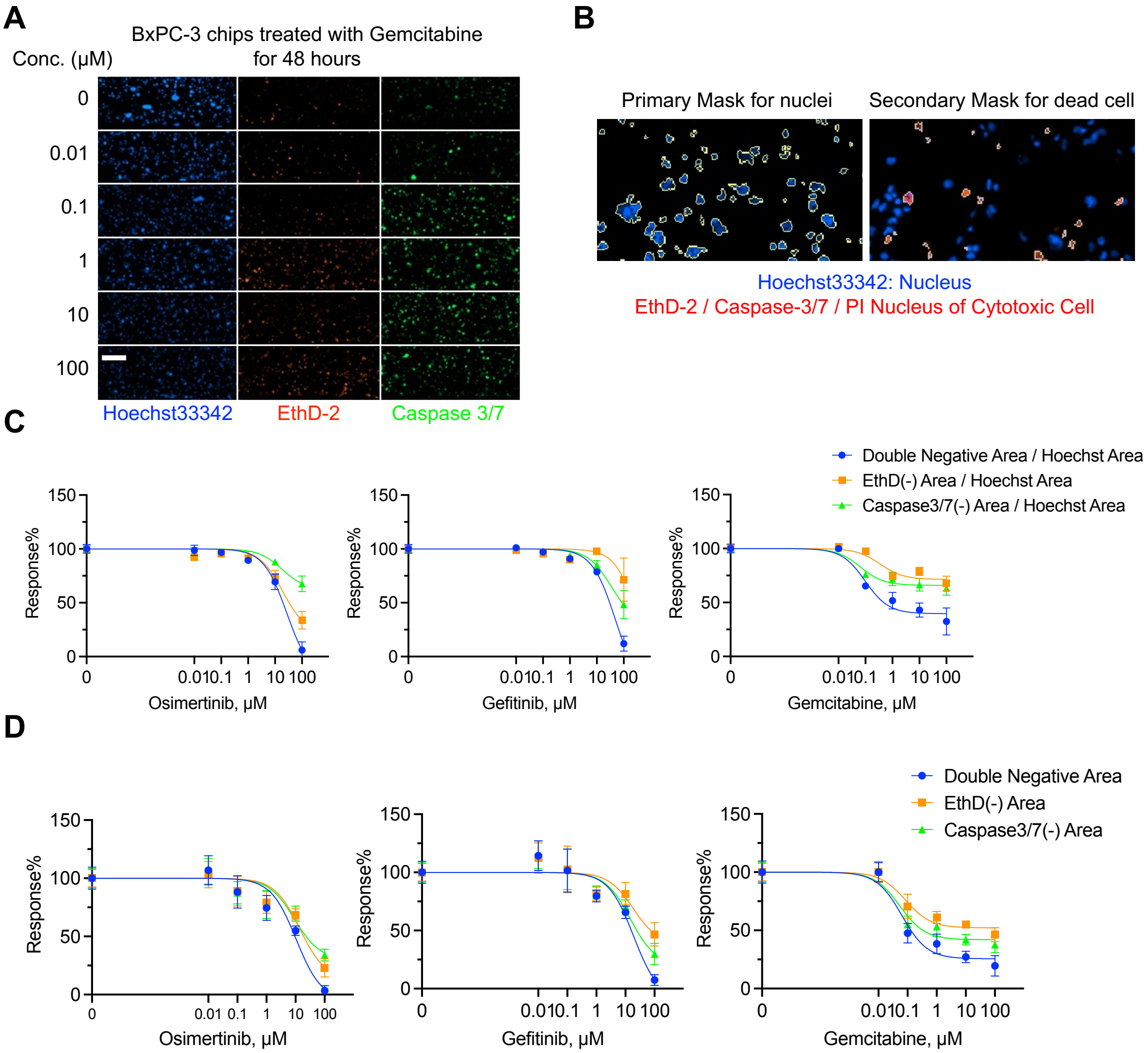

Supplementary Figure 7. Combining multiple staining on chip. (**A**) Representative images showing viability by Hoechst33342, EthD-2, and caspase 3/7 staining after drug treatment. Scale bar: 50 µm. (**B**) Schematic of the workflow for segmentation and downstream analysis of the triple-stained fluorescence images. (**C**) Normalized drug dose-response curves of BXPC-3 cells by using staining from EthD-2, caspase 3/7, or both. Data show mean ± s.d.; n=8 chips. (**D**) Similar to (**C**) but without normalization to the total Hoechst area. Data show mean ± s.d.; n=4 chips.

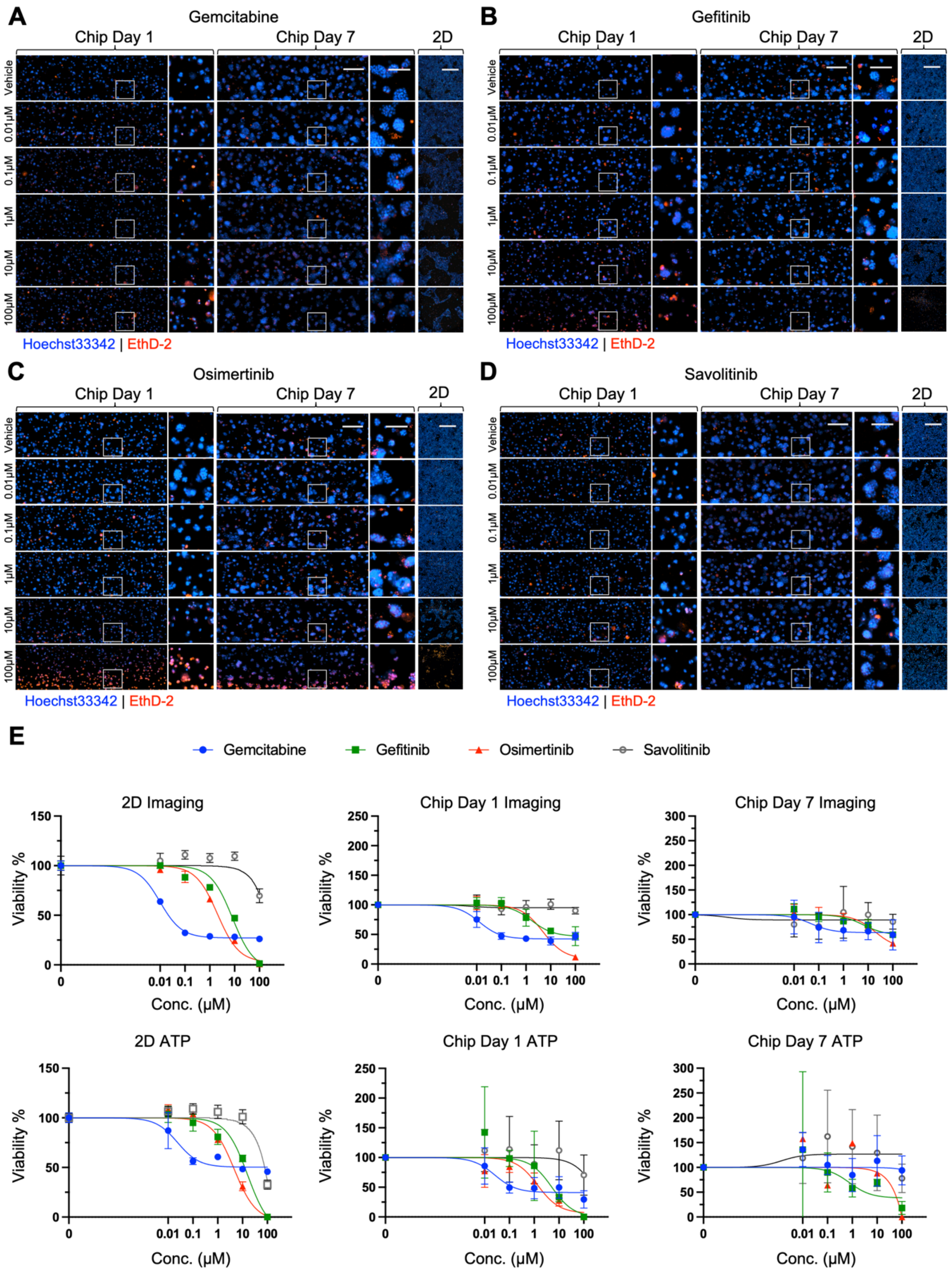

Supplementary Figure 8. Drug testing in pancreatic cancer chip. (**A**) Representative images

showing viability assessment by Hoechst33342 and EthD-2 staining after treatments with gemcitabine (**A**), gefitinib (**B**), osimertinib (**C**), and savolitinib (**D**) at indicated concentrations from 0.01 to 100 μM for 48 hours under 2D condition or after on-chip culture for 1 day or 7 days. Drug effects on BxPC-3 cell viability were analyzed using CellTiter-Glo ATP-assay or imaging detection (n=4). Scale bars represent 250, 100, 250 μm for chip, chip zoom-in, and 2D images, respectively. (**E**) The dose response curves were plotted. Data show mean ± s.d.; n=4 chips.

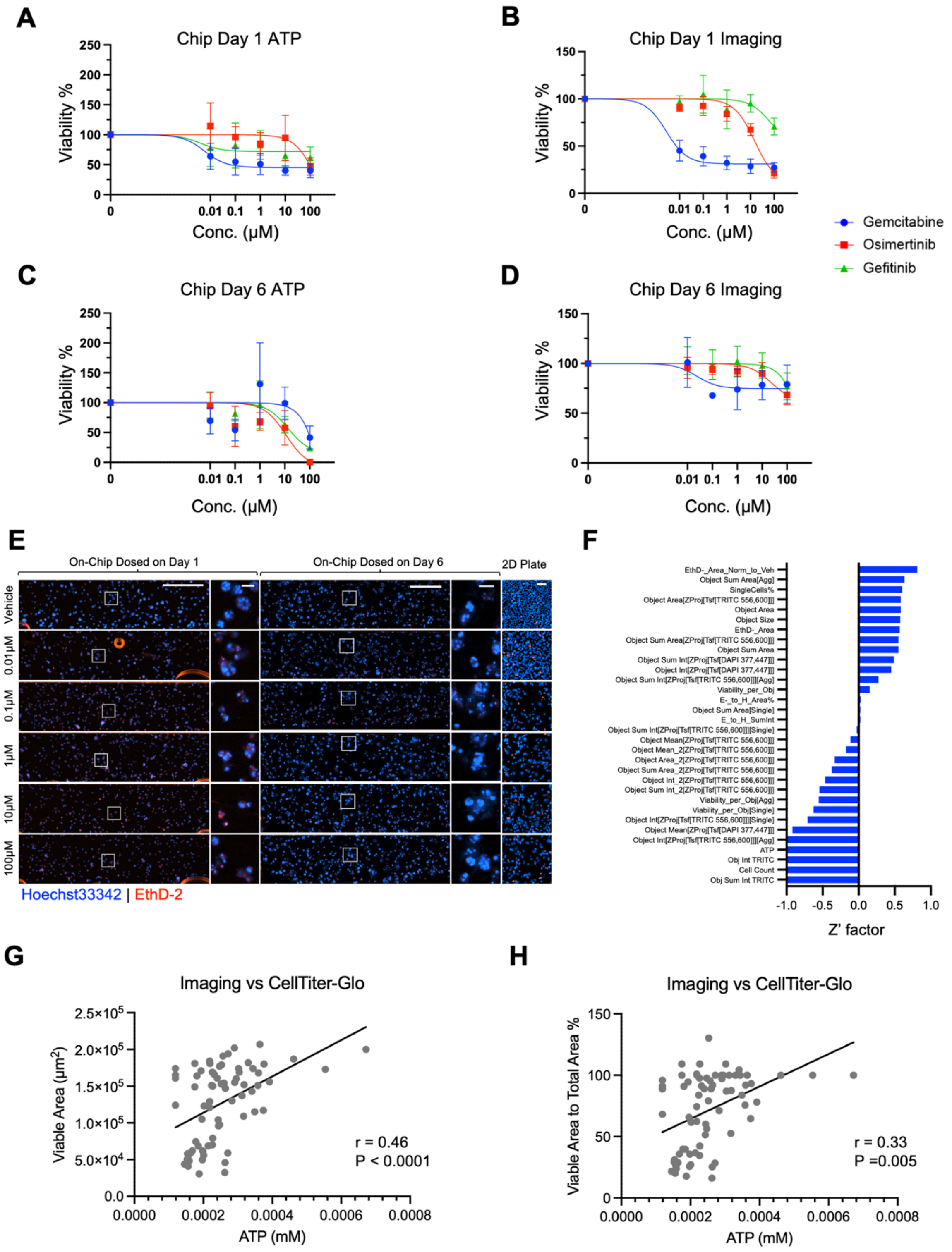

Supplementary Figure 9. Drug testing in lung cancer chip. (A-D) Dose-response curves of A549 cells treated with gemcitabine, osimertinib, or gefitinib after 1 day (**A,B**) or 6 days of culture (**C,D**) and analyzed using CellTitre-Glo (**A, C**) or imaging assay (**B,D**). Data show mean ± s.d.;

n=4 chips. **(E)** Representative images from gemcitabine-treated chips. Scale bars represent 500, 100, 100 μm for chip, chip zoom-in, and 2D images, respectively. (**F**) feature ranking by Z' score. (**G,H**) Correlations between the top 2 features and the CellTiter-Glo (ATP) results.

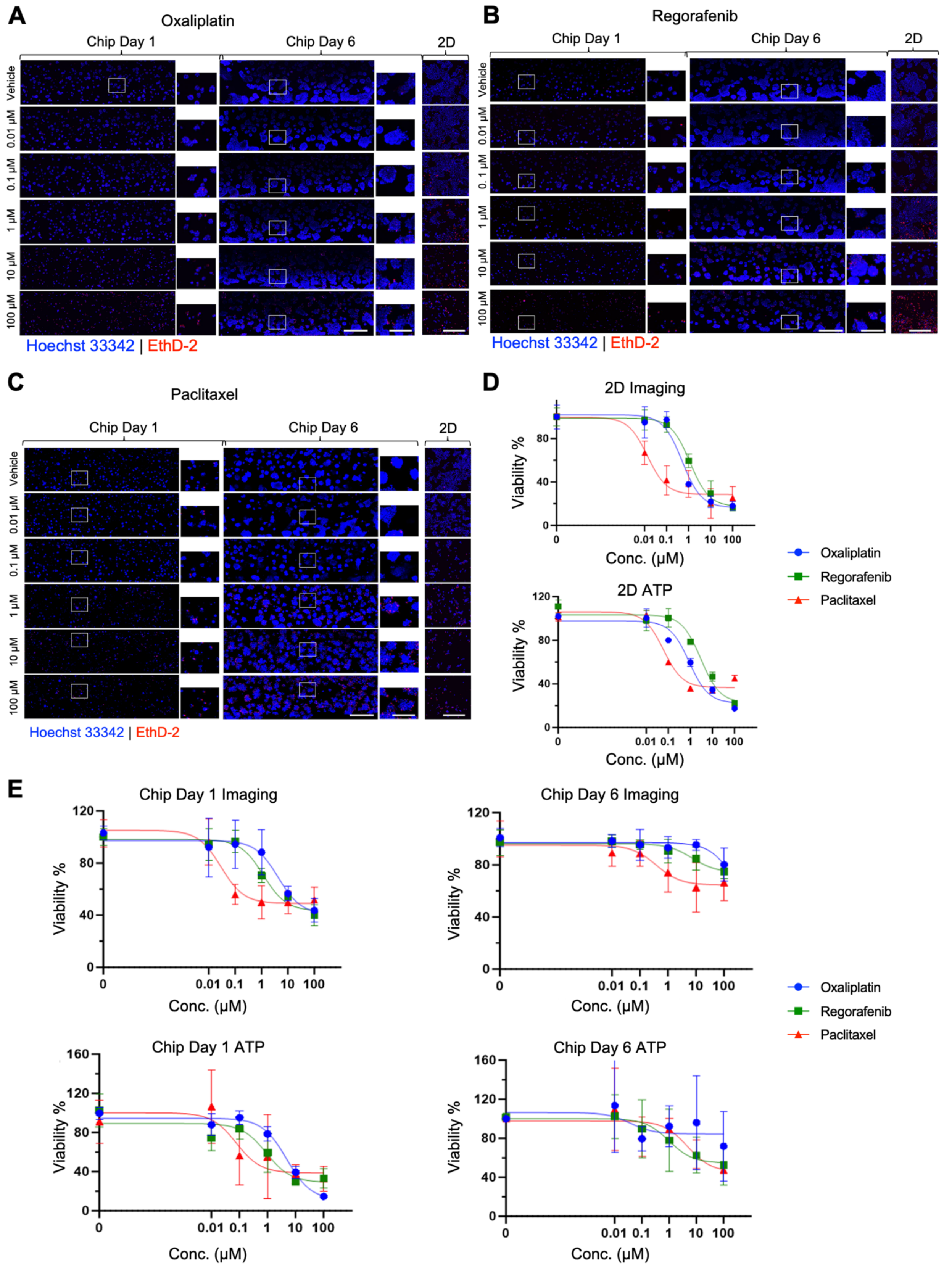

Supplementary Figure 10. Drug testing in colon cancer chip for HCT116. (**A**) Representative

images showing viability assessment by Hoechst33342 and EthD-2 staining after treatments with oxaliplatin (**A**), regorafenib (**B**), and paclitaxel (**C**) at indicated concentrations from 0.01 to 100 μM for 48 hours under 2D condition or after on chip culture for 1 day or 6 days. Scale bars represent 500, 200, 200 μm for chip, chip zoom-in, and 2D images, respectively. Drug effects on HCT116 cell viability were analyzed using CellTiter-Glo ATP assay or imaging detection (n=4). (**D**,**E**) The dose-response curves were plotted. IC_{50} and AUC values were calculated using the GraphPad Prism software. Data show mean ± s.d.; n=4–8 chips.

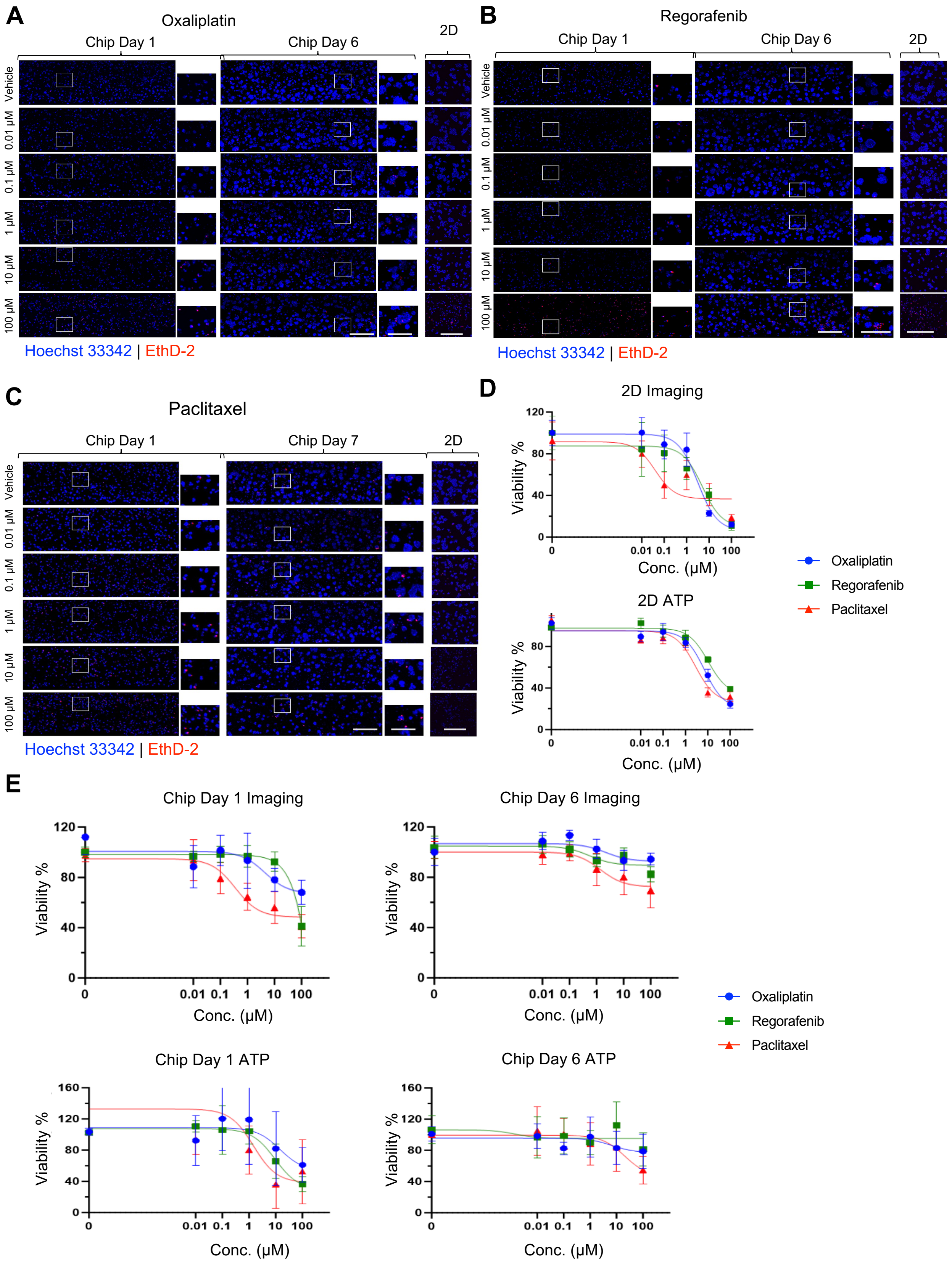

Supplementary Figure 11. Drug testing in colon cancer chip for HT-29. (A) Representative

images showing viability assessment by Hoechst33342 and EthD-2 staining after treatments with oxaliplatin (**A**), regorafenib (**B**), and paclitaxel (**C**) at indicated concentrations from 0.01 to 100 μM for 48 hours under 2D condition or after on chip culture for 1 day or 6 days. Scale bars represent 250, 100, 250 μm for chip, chip zoom-in, and 2D images, respectively. Drug effects on HT-29 cell viability were analyzed using CellTiter-Glo ATP assay or imaging detection. Data show mean ± s.d.; n=4 chips. (**D**, **E**) The dose response curves were plotted. IC_{50} and AUC values were calculated using the GraphPad Prism software. Data show mean ± s.d.; n=8 chips.

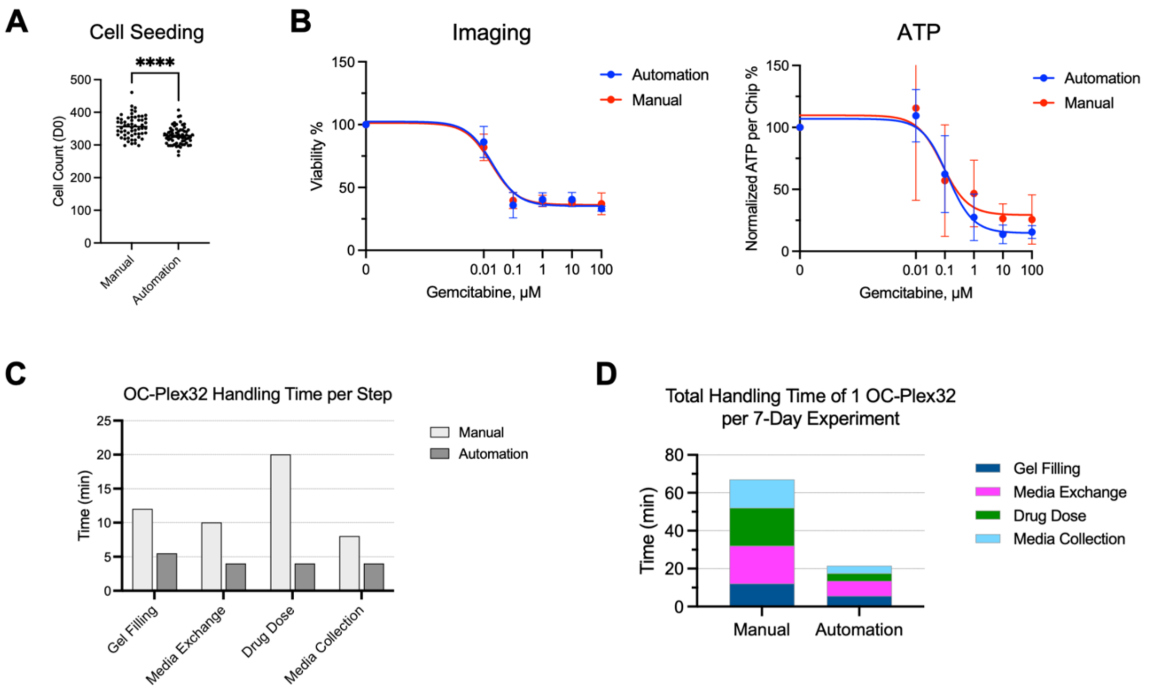

Supplementary Figure 12. Comparison of manual and automatic workflow. (**A**) Cell counts per chip for automated filling and manual filling. The standard deviations were 26.6 (automated) and 33.6 (manual), with coefficients of variation (%CV) of 8.11% and 9.45%, respectively. Data show mean ± s.d. Each point represents one chip (**B**) Dose response curves of A549 cells treated with Gemcitabine for 48 hours after cultured on chip for 1 day using automated and manual workflows. Drug effects were assessed by viability imaging (left) and CellTiter-Glo ATP assay (right). Data show mean ± s.d.; n=4 chips. (**C**) Process time per step working with OC-Plex32. (**D**) Total handling time for a 7-day experiment comparing automation and manual methods for 1 OC-Plex32 plate (32 chips).

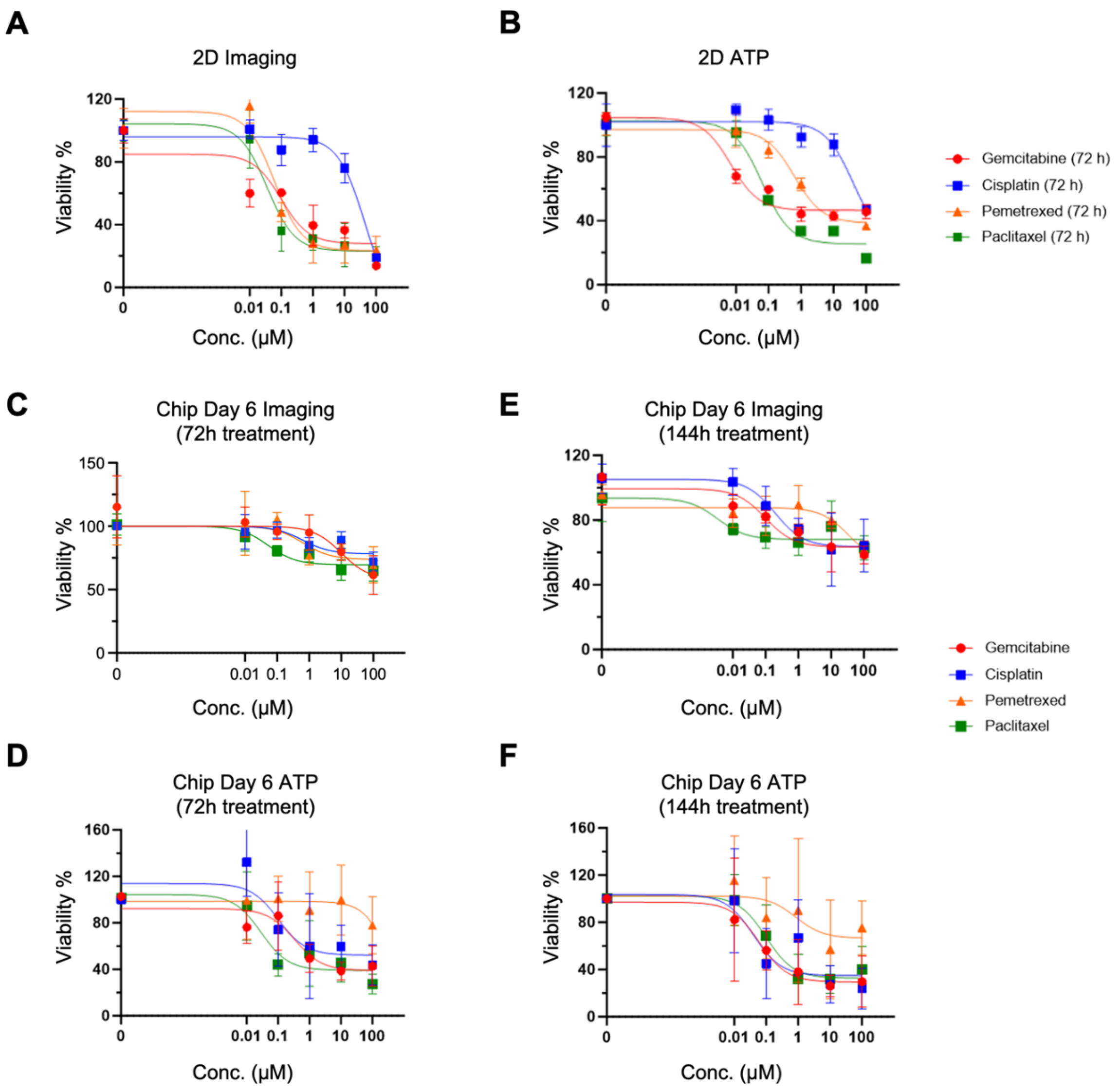

Supplementary Figure 13. Drug testing of primary cancer cells. After culturing for 6 days on OC-Plex devices or 1 day on 2D (**A**,**B**), primary lung cancer cells were treated with different concentrations of drugs (paclitaxel, gemcitabine, pemetrexed, cisplatin) for 72 h (**C**,**D**) or 144 h (**E**,**F**) on chip, and cell viability was detected by both imaging analysis and CTG assay. The IC_{50} and AUC values were calculated using the GraphPad Prism software. All data in this graph show mean ± s.d.; n=4–6 chips.

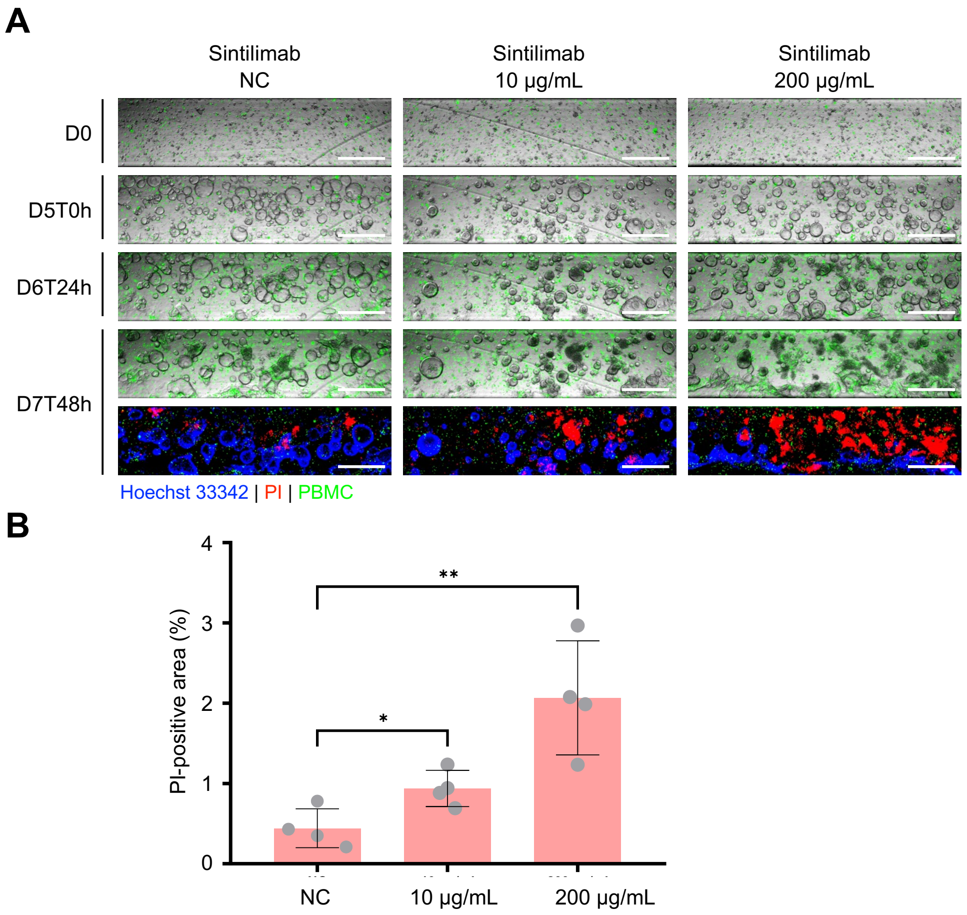

Supplementary Figure 14. Sintilimab exhibits dose- and time-dependent antitumor activity in the OC-plex microfluidic co-culture system. Following 5 days of co-culture with pre-activated PBMCs, gastric cancer organoids were treated with vehicle control, 10 μg/mL (low-dose), or 200 μg/mL (high-dose) of sintilimab. Bright-field or fluorescence images were acquired at 0, 24, and 48 h post-treatment. At 24 h, drug-treated organoids showed increased cell death and structural disintegration compared to the control. By 48 h, cytotoxicity was further enhanced, with greater organoid death in the high-dose group. PI/Hoechst staining further confirmed the dose-dependent antitumor effect of sintilimab, supporting the utility of this platform for preclinical drug evaluation. Nuclei: blue (Hoechst 33342); dead cells: red (PI); PBMCs: green (DiO). Scale bars: 500 μm. **(B)** Bar graph showing the percentage of PI-positive area within the ECM channel across different Sintilimab treatment groups. Data are presented as mean ± SD; n = 4 independent chips.

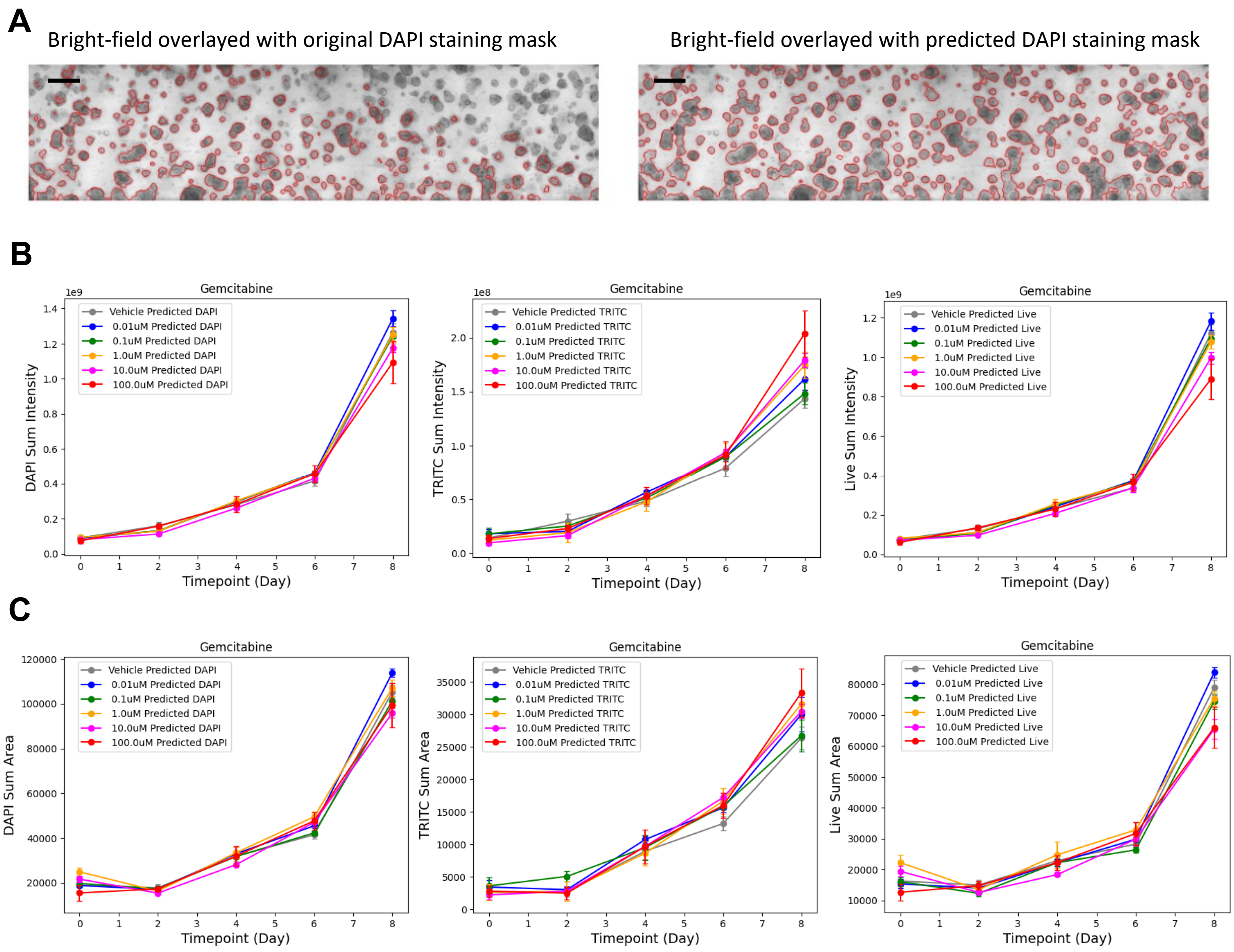

Supplementary Figure 15. Advantages of combing BF imaging and deep learning algorithms for assessing drug responses on chip. (**A**) Examples of in-silico staining overcome staining homogeneity issues with experimental staining. Scale bars: 165 µm. (**B**,**C**) Longitudinal tracking to measure sum intensities and sum areas across DAPI/TRITC/Live channels. Data show mean ± s.d.; N=4 chips.

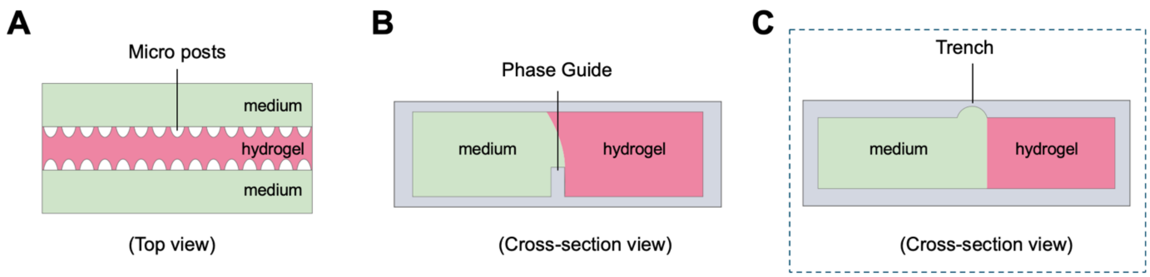

Supplementary Figure 16. Schematic illustration of common and our configurations in multi-channel OC devices. (**A**) Micropost-based separation; (**B**) phase-guide patterning; (**C**) our trench design combined with surface treatment.

Supplementary Table 1. Drug IC_{50} and AUC in different models by readout.

Cell Line	Drug	Model	Readout	IC_{50}	AUC
BXPC-3	Gemcitabine	2D	Imaging	0.01	2724
BXPC-3	Gefitinib	2D	Imaging	8.14	1712
BXPC-3	Osimertinib	2D	Imaging	2.04	1033
BXPC-3	Savolitinib	2D	Imaging	n.d.	8544
BXPC-3	Gemcitabine	Chip_day_1	Imaging	0.01	4251
BXPC-3	Gefitinib	Chip_day_1	Imaging	1.71	5054
BXPC-3	Osimertinib	Chip_day_1	Imaging	4.9	2247
BXPC-3	Savolitinib	Chip_day_1	Imaging	n.d.	9516
BXPC-3	Gemcitabine	Chip_day_7	Imaging	n.d.	6374
BXPC-3	Gefitinib	Chip_day_7	Imaging	6.44	6609
BXPC-3	Osimertinib	Chip_day_7	Imaging	19.25	5502
BXPC-3	Savolitinib	Chip_day_7	Imaging	n.d.	8929
BXPC-3	Gemcitabine	2D	CTG	0.02	5041
BXPC-3	Gefitinib	2D	CTG	16.06	2128
BXPC-3	Osimertinib	2D	CTG	4.64	1126
BXPC-3	Savolitinib	2D	CTG	n.d.	6797
BXPC-3	Gemcitabine	Chip_day_1	CTG	0.03	4127
BXPC-3	Gefitinib	Chip_day_1	CTG	5.18	1559
BXPC-3	Osimertinib	Chip_day_1	CTG	1.4	1188
BXPC-3	Savolitinib	Chip_day_1	CTG	n.d.	8509
BXPC-3	Gemcitabine	Chip_day_7	CTG	n.d.	9790
BXPC-3	Gefitinib	Chip_day_7	CTG	n.d.	4063
BXPC-3	Osimertinib	Chip_day_7	CTG	n.d.	5014
BXPC-3	Savolitinib	Chip_day_7	CTG	n.d.	12681
A549	Gemcitabine	Chip_day_1	CTG	0.0059	4072
A549	Osimertinib	Chip_day_1	CTG	n.d.	7259
A549	Gefitinib	Chip_day_1	CTG	n.d.	6430
A549	Gemcitabine	Chip_day_1	Imaging	0.003	2826
A549	Osimertinib	Chip_day_1	Imaging	22.97	4755
A549	Gefitinib	Chip_day_1	Imaging	n.d.	8389
A549	Gemcitabine	Chip_day_6	CTG	n.d.	7452
A549	Osimertinib	Chip_day_6	CTG	11.11	3249
A549	Gefitinib	Chip_day_6	CTG	13.34	4730
A549	Gemcitabine	Chip_day_6	Imaging	n.d.	7843
A549	Osimertinib	Chip_day_6	Imaging	23.16	8033
A549	Gefitinib	Chip_day_6	Imaging	n.d.	8871
HCT116	Oxaliplatin	2D	Imaging	0.51	1889
HCT116	Regorafenib	2D	Imaging	1.14	2172

HCT116	Paclitaxel	2D	Imaging	0.013	2871
HCT116	Oxaliplatin	2D	CTG	0.88	2556
HCT116	Regorafenib	2D	CTG	2.94	3129
HCT116	Paclitaxel	2D	CTG	0.059	3698
HCT116	Oxaliplatin	Chip_day_1	Imaging	4.24	4923
HCT116	Regorafenib	Chip_day_1	Imaging	1.16	4854
HCT116	Paclitaxel	Chip_day_1	Imaging	0.03	4619
HCT116	Oxaliplatin	Chip_day_1	CTG	4.78	3633
HCT116	Regorafenib	Chip_day_1	CTG	0.89	3298
HCT116	Paclitaxel	Chip_day_1	CTG	0.076	3052
HCT116	Oxaliplatin	Chip_day_6	Imaging	233	8767
HCT116	Regorafenib	Chip_day_6	Imaging	7.32	7852
HCT116	Paclitaxel	Chip_day_6	Imaging	0.38	6520
HCT116	Oxaliplatin	Chip_day_6	CTG	0.037	8458
HCT116	Regorafenib	Chip_day_6	CTG	0.97	5680
HCT116	Paclitaxel	Chip_day_6	CTG	4.68	5325
HT-29	Oxaliplatin	2D	Imaging	3.12	4155
HT-29	Regorafenib	2D	Imaging	4.97	5583
HT-29	Paclitaxel	2D	Imaging	7.86	3636
HT-29	Oxaliplatin	2D	CTG	6.99	2148
HT-29	Regorafenib	2D	CTG	1.35	2514
HT-29	Paclitaxel	2D	CTG	2.83	2362
HT-29	Oxaliplatin	Chip_day_1	Imaging	4.36	7451
HT-29	Regorafenib	Chip_day_1	Imaging	24.18	6961
HT-29	Paclitaxel	Chip_day_1	Imaging	0.35	4984
HT-29	Oxaliplatin	Chip_day_1	CTG	109	7461
HT-29	Regorafenib	Chip_day_1	CTG	107.6	5481
HT-29	Paclitaxel	Chip_day_1	CTG	132.9	4671
HT-29	Oxaliplatin	Chip_day_6	Imaging	2.19	9438
HT-29	Regorafenib	Chip_day_6	Imaging	0.54	9045
HT-29	Paclitaxel	Chip_day_6	Imaging	1.36	7584
HT-29	Oxaliplatin	Chip_day_6	CTG	95.86	8180
HT-29	Regorafenib	Chip_day_6	CTG	106.3	9709
HT-29	Paclitaxel	Chip_day_6	CTG	99.56	7099
Primary lung cancer	Gemcitabine	2D	Imaging	0.097	2662
Primary lung cancer	Cisplatin	2D	Imaging	45.87	5138
Primary lung cancer	Pemetrexed	2D	Imaging	0.059	2576
Primary lung cancer	Paclitaxel	2D	Imaging	0.0035	2370
Primary lung cancer	Gemcitabine	2D	CTG	0.0067	4187
Primary lung cancer	Cisplatin	2D	CTG	2.94	6978

Primary lung cancer	Pemetrexed	2D	CTG	0.86	4428
Primary lung cancer	Paclitaxel	2D	CTG	0.0062	2578
Primary lung cancer	Gemcitabine	Chip_day_6_72h_treatment	Imaging	6.44	7227
Primary lung cancer	Cisplatin	Chip_day_6_72h_treatment	Imaging	0.86	8137
Primary lung cancer	Pemetrexed	Chip_day_6_72h_treatment	Imaging	33.45	7758
Primary lung cancer	Paclitaxel	Chip_day_6_72h_treatment	Imaging	0.063	6589
Primary lung cancer	Gemcitabine	Chip_day_6_72h_treatment	CTG	0.33	4129
Primary lung cancer	Cisplatin	Chip_day_6_72h_treatment	CTG	0.1	5266
Primary lung cancer	Pemetrexed	Chip_day_6_72h_treatment	CTG	n.d.	8945
Primary lung cancer	Paclitaxel	Chip_day_6_72h_treatment	CTG	0.028	3763
Primary lung cancer	Gemcitabine	Chip_day_6_144h_treatment	Imaging	0.097	6183
Primary lung cancer	Cisplatin	Chip_day_6_144h_treatment	Imaging	0.19	6361
Primary lung cancer	Pemetrexed	Chip_day_6_144h_treatment	Imaging	36.75	7224
Primary lung cancer	Paclitaxel	Chip_day_6_144h_treatment	Imaging	0.0034	6949
Primary lung cancer	Gemcitabine	Chip_day_6_144h_treatment	CTG	0.13	1323
Primary lung cancer	Cisplatin	Chip_day_6_144h_treatment	CTG	0.039	2796
Primary lung cancer	Pemetrexed	Chip_day_6_144h_treatment	CTG	0.83	6718
Primary lung cancer	Paclitaxel	Chip_day_6_144h_treatment	CTG	0.096	3573
